# Intrinsic ROS Drive Hair Follicle Cycle Progression by Modulating DNA Damage and Repair and Subsequently Hair Follicle Apoptosis and Macrophage Polarization

**DOI:** 10.1155/2022/8279269

**Published:** 2022-07-14

**Authors:** Mingsheng Liu, Xiaomei Liu, Yuan Wang, Yutong Sui, Feilin Liu, Zinan Liu, Fei Zou, Kuiyang Zuo, Ziyu Wang, Wei Sun, Qi Xu, Dan Liu, Jinyu Liu

**Affiliations:** ^1^Department of Toxicology, School of Public Health, Jilin University, Changchun, China; ^2^Eye Center, The Second Hospital of Jilin University, Changchun, China; ^3^Department of Neuroscience, Mayo Clinic, Jacksonville, USA

## Abstract

Hair follicles (HFs) maintain homeostasis through the hair cycles; therefore, disrupting the hair cycle may lead to hair loss. Our previous study showed that apoptosis-inducing factor (AIF) nuclear translocation and poly [ADP-ribose] polymerase 1 (PARP1) upregulation induced apoptosis in mouse hair follicles during the hair cycle transition from anagen to catagen. However, the mechanism underlying this phenomenon remains unclear. In this study, we found that intrinsic ROS levels increased during the hair follicle cycle transition from anagen to catagen, followed by abrupt DNA breaks and activation of homologous recombinant and nonhomologous end joining DNA repair, along with the enhancement of apoptosis. Mice in different stages of the hair cycle were sacrificed, and the dorsal skins were collected. The results of western blot and histological staining indicated that AIF-PARP1 plays a key role in HF apoptosis, but their role in the regulation of the HF cycle is not clear. Mice were treated with inhibitors from anagen to catagen: treatment with BMN 673, a PARP1 inhibitor, increased DNA breaks and activated the cytochrome c/caspase-3-mediated apoptotic pathway, accelerating HF regression. Ac-DEVD-CHO (Ac), a caspase-3 inhibitor, attenuated HF degeneration by upregulating PARP1 expression, suggesting a seesaw relationship between cytochrome c-caspase-3- and AIF-PARP1-mediated apoptosis, wherein PARP1 may be the fulcrum. In addition, macrophages were involved in regulating the hair cycle, and the rate of M1 macrophages around HFs increased during catagen, while more M2 macrophages were found during anagen and telogen. Our results indicate that intrinsic ROS drive HF cycle progression through DNA damage and repair, followed by apoptosis. Intrinsic ROS drive hair follicle cycle progression by modulating DNA damage and repair, and consecutively, hair follicle apoptosis and macrophage polarization work together to promote the hair follicle cycle.

## 1. Introduction

Hair follicle (HF) is an appendage of the skin in mammals derived from the epidermal and mesenchymal interactions during embryonic development [1]. HF morphogenesis starts at an early embryonic stage and undergoes repetitive cycles of growth (anagen), apoptosis-driven regression (catagen), and relative quiescence (telogen) phases [2].

Reactive oxygen species (ROS) are a family of peroxides or free radicals mostly produced in the mitochondria through the electron transport chain during oxidative phosphorylation [3, 4]. Under physiological conditions, ROS function as a second messenger in the cells and transmit biological signals to regulate cell metabolism, proliferation, differentiation, and immune response [5]. ROS is necessary for the generation of hair follicles by promoting the proliferation of hair follicle cells [6]. However, the increasing evidence confirmed that clearance of ROS promoted hair follicle regeneration [7]. High ROS levels in hair follicle cells in catagen promote the degeneration of hair follicles via regulation of Trx1 and Foxp 1 [8]. However, the specific mechanism of ROS-induced hair follicle degeneration needs to be further explored. It is well known that excessive ROS may cause DNA damage or DNA breaks. Although eukaryotic cells have sophisticated DNA repair systems, and adverse effects such as aging [9], apoptosis [3], and malignancy [10] may occur when DNA damage exceeds the DNA repair capacity. DNA damage responsible for the regression of hair follicle and nonhomologous end joining (NHEJ) is activated to maintain genome integrity of hair follicle cells in aging mice [11]. Whether DNA damage and other repair pathway participate in the degeneration of hair follicle in young mice remains unclear.

PARP1 is a ribozyme that catalyses poly-ADP ribosylation in eukaryotic cells, which directs DNA repair through several strategies such as the base excision repair (BER), homologous recombination repair (HR), and NHEJ pathways to maintain genome stability in eliminating DNA breaks [12–14]. Moreover, ability of cells to repair DNA damage is limited, and PARP1 overactivation during DNA repair inevitably induces PAR accumulation, resulting in AIF nuclear translocation, subsequently leading to large DNA fragmentation and finally apoptosis [15]. In addition, as a substrate of caspase-3, PARP1 is cleaved into an 89 kDa PARP1 cleavage fragment, and the 89 kDa fragment serves as a cytoplasmic PAR carrier to induce AIF-mediated apoptosis [16, 17]. Therefore, PARP1 displays a double-edged sword, participating in DNA repair and cellular apoptosis. Apoptosis-driven catagen is considered a critical period for driving the hair cycle progression [18]. Increasing evidence suggests that caspase-dependent apoptosis signalling pathways play an important role in hair cycle transition from the anagen to catagen phase [18, 19]. Our recent study showed that cyclosporine A increases hair follicle growth by suppressing AIF nuclear translocation and PARP1 expression, suggesting that the AIF-PARP1-mediated apoptotic pathway, a caspase-independent apoptotic signalling pathway, may be involved in HF transition from anagen to catagen [20]. However, the roles of AIF-PARP1 and cytochrome c- (Cyto c-) caspase-3 (Cas 3) signalling pathway in hair cycle transition from anagen to catagen and their temporal and spatial regulation are unclear.

To maintain homeostasis, clearance of apoptosis cells is a fundamental process in which macrophages play important roles [21]. Macrophages identify and clear apoptotic cells by efferocytosis and participate in tissue repair and regeneration via M1/M2 phenotype polarization [21–23]. It has been reported that ROS, DNA damage, and the activation of PARP1 participate in the recruitment and polarization of macrophages in tissues [24, 25]. When macrophages are polarized from M1 to M2 type, they promote resident tissue cells to release growth factors, such as VEGF, EGF, or FGF, to enhance tissue regeneration [26]. The polarization of macrophages from M1 to M2 during the transition of the hair cycle from anagen to late catagen has been reported [27]. The presence of macrophages is necessary for hair follicle to enter anagen by stimulating the proliferation of hair follicle cells; M2 macrophages promote hair neogenesis [28]. However, changes in macrophage polarization during the complete hair cycle in vivo and the underlying mechanisms need to be further explored.

In this study, we explore the consequence of ROS-mediated DNA damage and repair along with the spatial-temporal relationship between Cyto c/Cas 3- and AIF-PARP1-mediated cellular processes during various phases of the HF cycle. We found that ROS levels increased, followed by DNA damage and repair and Cyto c/Cas 3- and AIF-PARP1-mediated apoptosis activation during hair cycle transition from anagen to catagen. A seesaw relationship exists between Cyto c/Cas 3- and AIF-PARP1-mediated apoptosis, wherein PARP1 may be the fulcrum.

## 2. Materials and Methods

### 2.1. Animals

All experiments were approved by the Medical Ethics Committee of Jilin University School of Public Health. C57BL/6 mice (purchased from Beijing Hua Fu Kang Biological Technology Co. Ltd., China) were housed and bred at the Laboratory Animal Center of Jilin University, China. The offspring obtained on postnatal days 11 (P11, anagen), 16 (P16, early catagen), 18 (P18, late catagen), and 21(P21, telogen) were sacrificed by cervical dislocation, and the dorsal skin was collected after shaving the hair.

### 2.2. Treatment of Mice with the PARP1 Inhibitor BMN 673 and the Caspase-3 Inhibitor Ac-DEVD-CHO

To compare the role of AIF-PARP1 and Cyto c/Cas 3 apoptotic signalling pathways in the degeneration of hair follicles, littermates of C57BL/6 mice (6/group) were administered daily with the PARP1 inhibitor (BMN 673, MCE, USA) or Cas 3 inhibitor (Ac-DEVD-CHO, Selleck, USA) or a combination of both, through intraperitoneal injection, from postnatal days 11 to 17 at the dosage indicated in the results. The body weight of the individual mouse in each group was monitored to evaluate the potential toxicity of the above-mentioned inhibitors. Mice were sacrificed via cervical dislocation on postnatal day 18, and the dorsal skin was collected from each mouse. HE staining was performed to detect histological changes in the hair follicle following treatment with the PARP1 inhibitor or (and) Cas 3 inhibitor. Western blotting and immunofluorescence staining were performed to detect DNA damage, repair, and apoptosis in the hair follicles.

### 2.3. HE Staining

Full-thickness dorsal skin of the mice was collected and fixed in formalin (Dingguo, Beijing, China), embedded in paraffin, and cut into 5 *μ*m thick sections. After rehydration, the skin sections were stained with haematoxylin and eosin and observed under a microscope (DMI 4000 B, Leica, Germany). Skin thickness (distance from the epidermis to the subcutaneous muscle layer) and hair bulb diameter (diameter of the most expansive part of the bulb) were measured using ImageJ. Three fields were randomly selected under the microscope to count the number of HFs, and the mean value was calculated as the HF density.

### 2.4. Detection of Intrinsic ROS in HFs

Full-thickness dorsal skin was collected, embedded in OCT, and cut into 10 *μ*m thick sections. The frozen sections were incubated with CM-H2DCFDA (Life, New York, USA) at 37°C for 30 min to detect the ROS. Nuclei were stained with DAPI for 10 min. Images were captured using a microscope (DMI 4000 B, Leica, Germany), and the mean fluorescence density (MFI) was analysed using ImageJ.

### 2.5. Protein Extraction and Western Blotting

The dorsal skin was collected after subcutaneous fat stripping and was divided into three parts. One part was homogenized in RIPA buffer (Beyotime, China) containing PMSF to extract total protein content. The second part was used to extract total nuclear protein using the method described by Dignam et al. [29]. Briefly, the skin was homogenized with a tissue homogenizer in an ice water bath, 10 s each time, 30 s apart, and nuclei were separated by centrifugation at 800 × *g* for 10 min. The nuclei were resuspended and lysed with lysis buffer to harvest the nuclear protein. The third part was used to extract mitochondrial proteins using a mitochondrial protein extraction kit (KeyGEN BioTECH, China) according to the manufacturer's instructions. Briefly, the skin was homogenized and centrifuged for 5 min at 800 × *g*, and the supernatant was transferred to medium buffer and centrifuged for 10 min at 15,000 × *g*. Then, the precipitate was resuspended and lysed with lysis buffer.

The protein samples were separated by 8-10% SDS-PAGE and then transferred onto PVDF membranes (Millipore, USA). The membranes were blocked with 5% skim milk and then incubated with various antibodies, as indicated in the respective figures and listed in Supplementary Table S1, at 4°C overnight. The next day, the membranes were washed with TBST, incubated with the corresponding secondary antibodies (anti-mouse antibody, anti-rabbit antibody, and anti-goat antibody, Proteintech, China), and visualized using an ECL detection system (Tanon 5200, Tanon, China). The grayscale intensities of the protein bands were analysed using the Tanon Gis analytical software (Tanon, China).

### 2.6. Immunohistochemistry and Immunofluorescence Staining

After deparaffinization and rehydration, the sections were blocked with 1% BSA and incubated at 4°C overnight with various antibodies, as indicated in the respective figures and listed in Supplementary Table S2. The next day, the sections were washed with PBS thrice. For immunofluorescence, the sections were incubated with fluorescent-labelled secondary antibodies listed in Supplementary Table S2 for 1 h at 25°C, and nuclei were stained with DAPI. For immunohistochemistry, samples were incubated with horseradish peroxidase-labelled secondary antibody (Maixin, China) for 10 min. After a PBS wash, samples were incubated with DAB reagent (Maixin, China) for 10 min. Images were captured using a microscope (DMI 4000 B, Leica, Germany) and analysed using ImageJ.

For cryosection, full-thickness dorsal skin was collected, embedded in OCT, and cut into 5 *μ*m thick sections. The cryosections were incubated with anti-57 kDa AIF antibodies (dilution 1 : 50, Santa Cruz, USA) or anti-Ki67 antibody (dilution 1 : 200, Abcam, USA) overnight at 4°C. The next day, sections were washed with TBS and incubated with Alexa Fluor 594-conjugated anti-mouse IgG (dilution 1 : 1000, CST, USA) or Alexa Fluor 594-conjugated anti-rabbit IgG (dilution 1 : 1000, CST, USA). After the nuclei were stained with DAPI, the sections were mounted using an antifade mounting gel. Images were captured using a microscope (Leica, DMI 4000 B, Germany) and analysed using ImageJ.

### 2.7. TUNEL Staining

Apoptosis was detected by the terminal deoxynucleotidyl transferase-mediated dUTP nick end labelling (TUNEL) assay using the DeadEnd Fluorometric TUNEL assay kit (Promega, USA). The paraffin sections (dewaxed and rehydrated) or frozen sections were permeabilized with 0.1% Triton X-100 in TBS for 30 min at 25°C. After a TBS wash, sections were incubated with 50 *μ*l Nucleotide Mix (with terminal deoxynucleotidyl transferase) at 37°C in the dark for 1 h. Next, 2× SSC (saline sodium citrate) was added to stop the enzymatic reaction. Nuclei were stained with DAPI for 10 min after which the sections were sealed with an antifade mounting gel, and pictures were captured with a microscope (Leica, DMI 4000 B, Germany) and analysed using ImageJ.

### 2.8. Detection of NAD+ and NADH

NAD level, which consists of NAD+ and NADH, in dorsal skin of the mice from different hair cycle stages was measured using an assay kit, according to the manufacturer's instructions (Beyotime Technology, China). To measure NAD concentrations, 30 mg skin tissue of each sample was homogenized, and the supernatant was collected following centrifugation. 20 *μ*l of each sample was mixed with 90 *μ*l alcohol dehydrogenase working solution and incubated at 37°C for 10 min in the dark to convert NAD+ to NADH. Subsequently, 10 *μ*l colour developing solution was added in and incubated at 37°C in the dark for 30 min. Absorbance was measured at 450 nm via a microplate reader (Bio-Tek, USA). For NADH detecting, samples were heated at 60°C in the PCR instrument for 30 min to decompose NAD+ prior to detection.

### 2.9. Statistical Analysis

Statistical analyses were performed using GraphPad Prism 8.02. The data is expressed as the mean ± standard deviation. Multiple comparisons were made with a one-way analysis of variance (ANOVA), and statistical significance was set at *P* < 0.05.

## 3. Results

### 3.1. HFs Degenerated with Hair Cycle Progress

HE staining showed that starting from early catagen, HF density and hair bulb diameter decreased with hair cycle progression, reaching a valley in telogen, consistent with decreased skin thickness (Figure S1). Ki67-positive cells were mainly distributed in the outer root sheath and hair bulb matrix, displaying the highest mean fluorescence intensity (MFI) in the anagen phase (*P* < 0.001). After that, the MFI abruptly decreased until it was undetectable in the late catagen and telogen phases (Figure S2).

### 3.2. Intrinsic ROS-Mediated AIF-PARP1 and Cyto c/Caspase-3 Apoptotic Pathways Participated in Hair Cycle Progression

**Figure 1:**
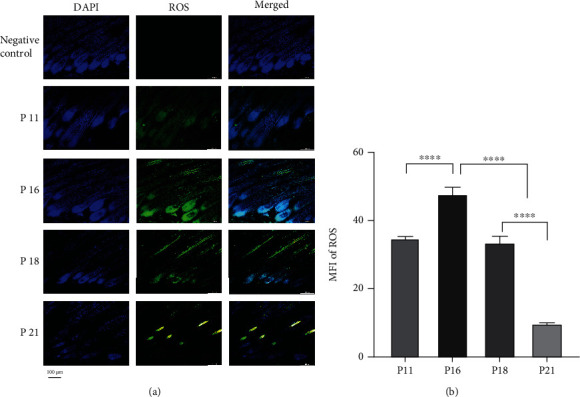
Level of ROS in hair follicles was elevated in catagen in dorsal skin sections, which were obtained from mice at different stages of the hair cycle (postnatal days), as indicated, and were processed for histology and immunohistochemistry, as described in Materials and Methods. (a) ROS was widely distributed in HFs. (b) Mean fluorescence intensity (MFI) of ROS in HFs was elevated in early catagen, while it decreased in telogen. ^∗∗∗∗^*P* < 0.0001. Scale bar (a): 100 *μ*m.

ROS is known to regulate cell metabolism, proliferation, differentiation, and immune response during physiological conditions, and hair follicles undergo apoptosis during hair cycle transition from anagen to catagen. Therefore, we measured kinetic ROS levels during this transition. Our results showed that ROS MFI significantly increased and reached a peak in the early catagen phase (*P* < 0.0001), which was significantly higher than that in the anagen phase (Figure 1). After that, ROS level decreased and reached a valley in the telogen phase, which was significantly lower than that in the anagen and catagen phases (Figure 1). Similar to the kinetic changes in ROS levels, TUNEL staining showed that apoptotic cells were extensively distributed in the outer root sheath and hair bulb in the anagen and early catagen stages, with the highest MFI in early catagen with a fewer number of apoptotic cells found in telogen (Figure S2). The results suggested that intrinsic ROS may participate in the apoptotic process of hair cycles.

**Figure 2:**
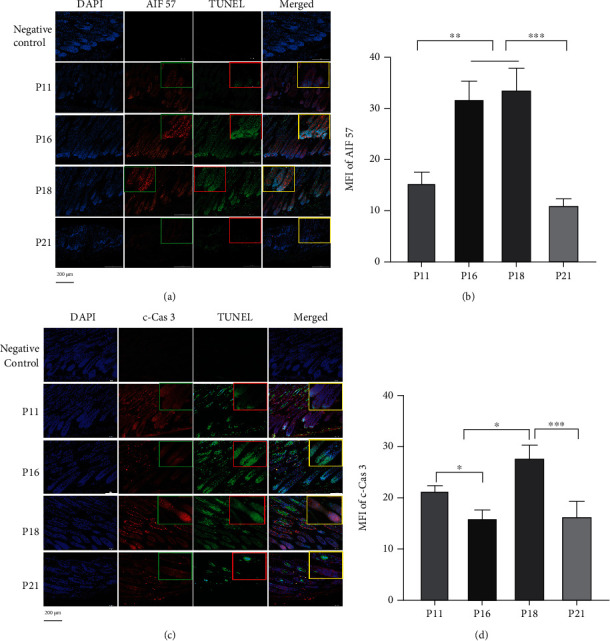
Nuclear translocation of AIF and activation of caspase-3 were consistent with the cell death in HFs. (a, b) 57 kDa AIF translocated into the nucleus in TUNEL-positive cells, and MFI of 57 kDa AIF was enhanced in catagen consistent with TUNEL. (c, d) Cleaved caspase-3 (c-Cas 3) was activated in TUNEL-positive cells during the hair cycle, while MFI of c-Cas 3 was decreased in early catagen and elevated in late catagen. The insert boxes in the figure are partially enlarged pictures. Horizontal line in the statistical analysis graph means that each of the groups under the line was statistically different compared with another group. ^∗^*P* < 0.05, ^∗∗^*P* < 0.01, and ^∗∗∗∗^*P* < 0.0001. Scale bar (a, c): 200 *μ*m.

**Figure 3:**
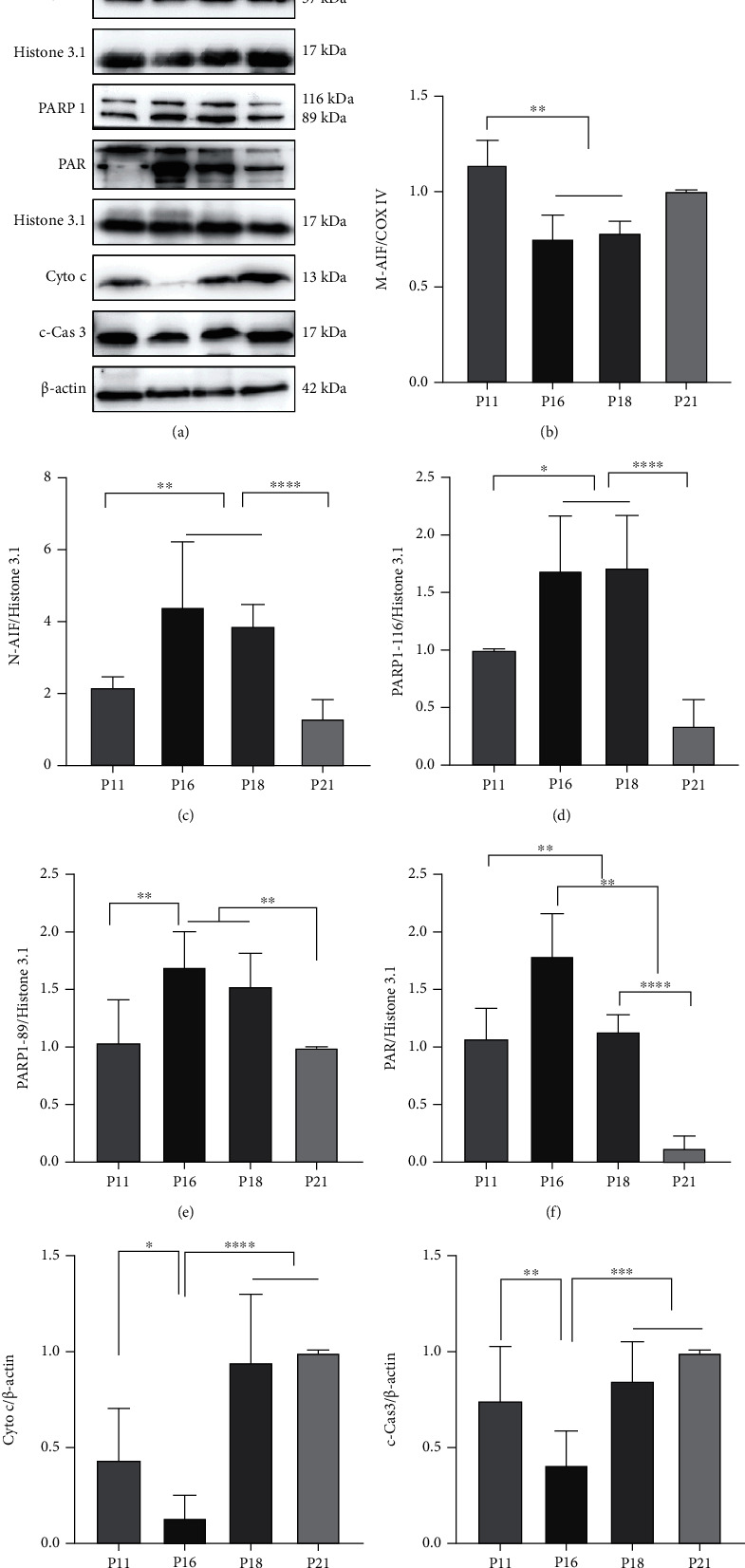
Apoptosis was involved in regulating the hair cycle. (a–h) Western blot (*n* = 6) showed that capacity of PARP1 was enhanced in catagen that expressions of 116 kDa PARP1 and PAR were increased. The level of 89 kDa PARP1 was increased in catagen, and more AIF translocated from mitochondria to the nucleus in HFs in catagen. Cas 3 was activated in the hair follicle during the whole hair cycle, while c-Cas 3 was downregulated in early catagen with Cyto c. ^∗^*P* < 0.05, ^∗∗^*P* < 0.01, ^∗∗∗^*P* < 0.001, and ^∗∗∗∗^*P* < 0.0001.

ROS induces mitochondria-mediated apoptosis, with both Cyto c/Cas 3 and AIF-PARP1 participating in mitochondria-mediated apoptosis. Therefore, we performed immunofluorescence staining to analyse the kinetic distribution of Cyto c/Cas 3 and AIF-PARP1 apoptosis signalling pathways in hair cycles. Results showed punctate staining of 57 kDa AIF distributed in the nuclei of HFs in early and late catagen phases and exhibited elevated MFI (*P* < 0.01), consistent with the MFI of tunnel staining (Figures 2(a) and 2(b), Figure S3). Immunofluorescence staining showed that the MFI of cleaved Cas 3 (c-Cas 3) was elevated in the late catagen phase (*P* < 0.05) (Figures 2(c) and 2(d)). Western blotting showed that during hair cycle transition from the anagen to catagen phase, the expression of 62 kDa AIF was downregulated (*P* < 0.01) (Figures 3(a) and 3(b)). From catagen to telogen, however, 62 kDa AIF expression was upregulated, reaching the level seen in the anagen phase. In contrast, 57 kDa AIF expression was upregulated from anagen to early and late catagen (*P* < 0.01), was downregulated from catagen to telogen (*P* < 0.0001), and was even lower than that in anagen (Figures 3(a) and 3(c)). In agreement with the 57 kDa AIF expression, the 116 kDa PARP1 expression was dramatically upregulated from anagen to early and late catagen (*P* < 0.05) and downregulated from catagen to telogen (*P* < 0.0001) (Figures 3(a) and 3(d)). The expression of 89 kDa PARP1 increased from anagen to early and late catagen (*P* < 0.01) and decreased from catagen to telogen (*P* < 0.01) (Figures 3(a) and 3(e)). As PARP1 catalyses the transfer of ADP-ribose units onto target proteins and the majority of PAR polymer synthesis derives from PARP1, we detected PAR expression in HFs. Western blotting showed that PAR was remarkably upregulated during hair cycle transition from anagen to catagen (*P* < 0.01), reaching a peak at the early catagen phase, and was downregulated with the progression of the HF cycle (Figures 3(a) and 3(f)). Cyto c and c-Cas 3 expression was downregulated in HFs from anagen to catagen (*P* < 0.05). After that, their expression was upregulated in late catagen and telogen, where it was even higher than that in the anagen phase (Figures 3(a), 3(g), and 3(h)).

### 3.3. PARP1-Mediated DNA Damage and Repair Participated in Hair Cycle Transition from Anagen to Early Catagen

**Figure 4:**
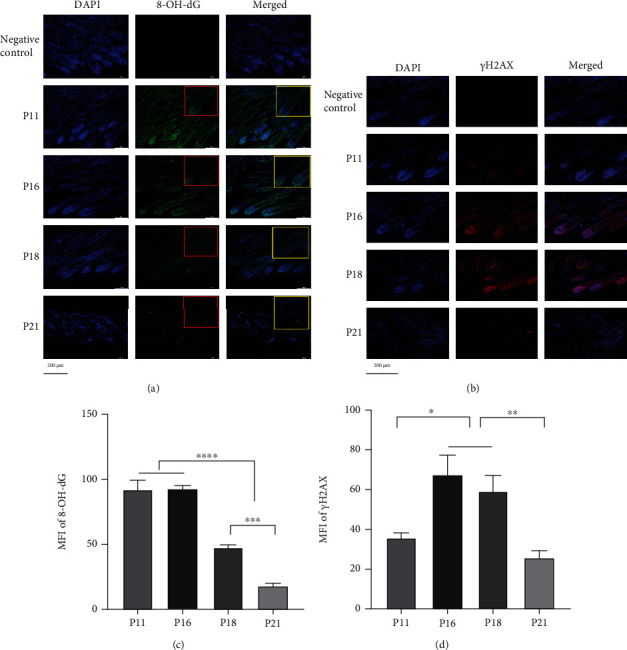
Expression and distribution of DNA damage markers in hair follicles during the hair cycle. (a, c) Expression of 8-OH-dG in HFs was decreased in late catagen and telogen. (b, d) *γ*H2AX was expressed mostly in bulbs in anagen and extended upward in catagen. MFI of *γ*H2AX was upregulated in catagen. ^∗^*P* < 0.05, ^∗∗^*P* < 0.01, and ^∗∗∗^*P* < 0.001. Scale bar (a, b): 200 *μ*m.

**Figure 5:**
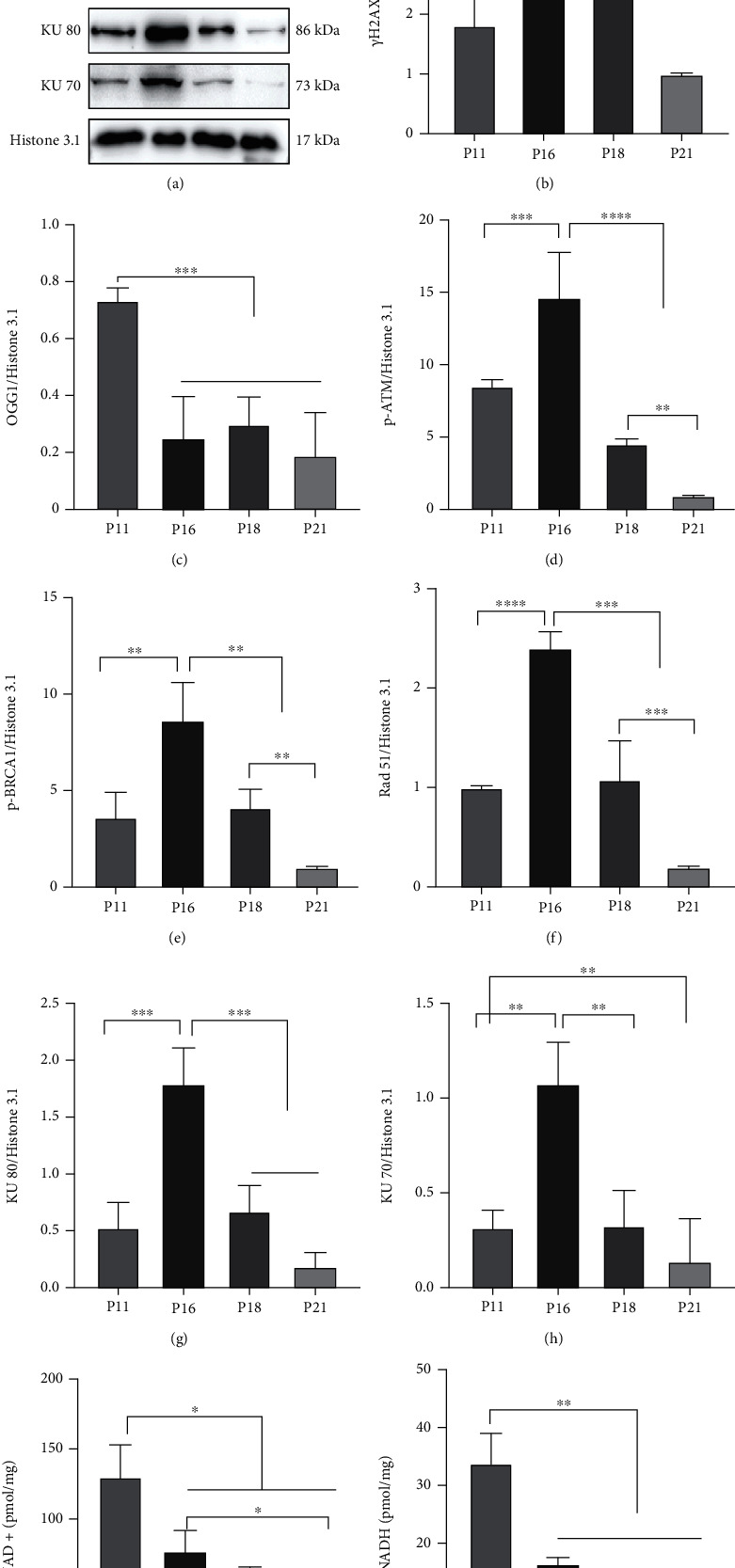
DNA repair was involved in regulating the hair cycle. (a–h) Western blot showed that expression of *γ*H2AX was increased in catagen. DNA damage repair proteins of HR and NHEJ pathways, p-ATM, p-BRCA 1, Rad51, KU80, and KU70, were upregulated in early catagen, while the DNA damage repair protein of the BER pathway—OGG1—was decreased during catagen and telogen. (i, j) Concentrations of NAD+ and NADH in HFs were decreased in catagen and telogen. ^∗^*P* < 0.05, ^∗∗^*P* < 0.01, ^∗∗∗^*P* < 0.001, and ^∗∗∗∗^*P* < 0.0001.

**Figure 6:**
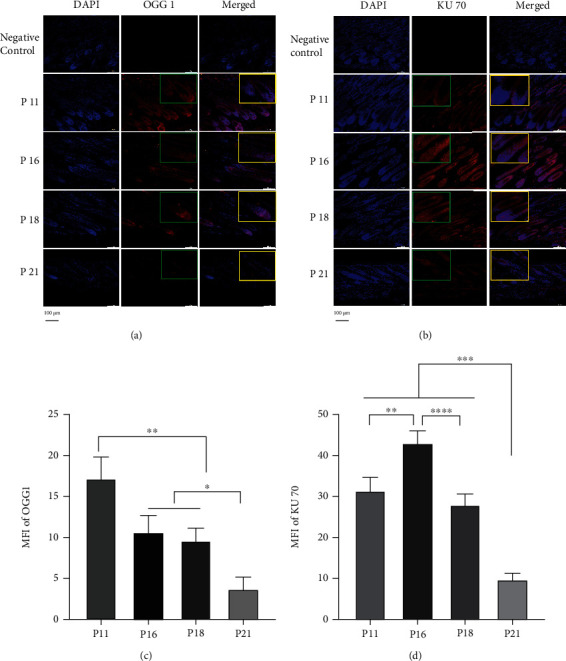
Activity of DSB repair in HFs strengthened in early catagen, while activity of SSB repair weakened in early catagen. (a, c) Immunofluorescence staining showed that OGG1 was distributed in HFs, while the MFI of OGG1 was decreased in catagen and telogen. (b, d) Immunofluorescence staining showed that KU70 was distributed widely in HFs, and the expression of KU70 was enhanced in early catagen. ^∗^*P* < 0.05, ^∗∗^*P* < 0.01, ^∗∗∗^*P* < 0.001, and ^∗∗∗∗^*P* < 0.0001. Scale bar (a, b): 100 *μ*m.

Increased ROS levels induce DNA damage, and PARP1 participates in DNA repair. Therefore, we analysed DNA single-streak and double-streak breaks as well as DNA repair-related pathways, including BER, HR, and NHEJ, by immunofluorescence staining and western blot assay. Immunofluorescence staining showed that 8-OH-dG, indicating single-streak DNA breaks, displayed similar MFI in anagen as well as in early catagen. After that, the MFI significantly decreased (*P* < 0.0001), dropping to the lowest level at the telogen phase (*P* < 0.001) (Figures 4(a) and 4(c)), whereas the MFI of *γ*H2AX, indicating double-streak DNA breaks, abruptly increased in the early and late catagen stages (*P* < 0.05, anagen vs. early catagen) and decreased in telogen to the same level as that in anagen (Figures 4(b) and 4(d)). Consistent with immunofluorescence staining, the western blot assay showed that *γ*H2AX expression was significantly upregulated, reaching peak in the early catagen phase (*P* < 0.05), and slightly downregulated in the late catagen phase, but still higher than that in the anagen phase (*P* < 0.05). With hair cycle progression, however, *γ*H2AX expression further decreased, even lower than that in the anagen phase (*P* < 0.05) (Figures 5(a) and 5(b)). To explore the HF functionality in repairing DNA damage caused by intrinsic ROS, we monitored PARP1-mediated DNA repair proteins related to BER, HR, and NHEJ. Western blotting and immunofluorescence staining showed that OGG1 expression was significantly downregulated in the early catagen phase compared with anagen (*P* < 0.001) and remained low until telogen (*P* < 0.01) (Figures 5(a), and 5(c), Figures 6(a), and 6(c)). Interestingly, the expression of DNA repair proteins related to HR and NHEJ, such as p-ATM, p-BRCA 1, Rad51, and KU70/80, was dramatically upregulated in the early catagen phase, reaching a peak, which was significantly higher than that in the anagen (*P* < 0.01), late catagen (*P* < 0.01), and telogen phases (*P* < 0.01) (Figures 5(a), and 5(d)–5(h), Figures 6(b), and 6(d)). As the substrate of PARP1 during PAR formation, NAD+ was consumed, and hence, the concentrations of NAD+ and NADH were decreased in catagen and telogen (*P* < 0.05) (Figures 5(i) and 5(j)).

### 3.4. Inhibition of PARP1 Activity Attenuated DNA Repair and Activated Cyto c/Caspase-3-Mediated Apoptotic Signalling Pathway

**Figure 7:**
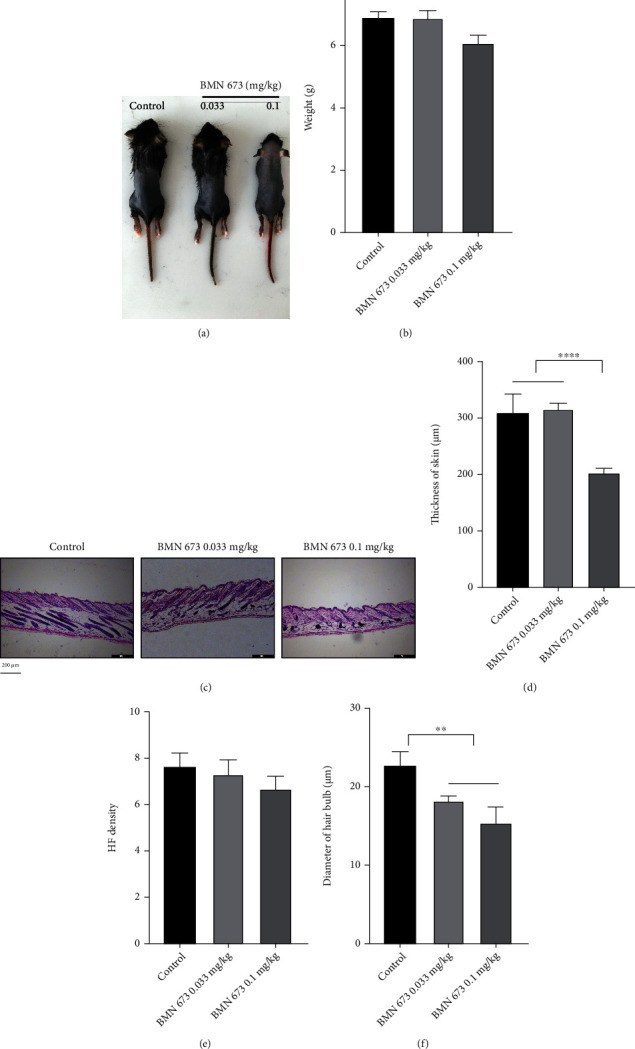
PARP1 inhibition promoted HF degeneration. (a) Gross observation of mice treated with BMN 673. (b) Weight loss in mice treated with BMN 673 at 0.1 mg/kg. (c–f) HE staining of the dorsal skin showed that the skin was thinned in mice treated with BMN 673 0.1 mg/kg and diameters of bulbs were reduced in mice treated with BMN 673. ^∗^*P* < 0.05, ^∗∗^*P* < 0.01, and ^∗∗∗^*P* < 0.001. Scale bar (c): 200 *μ*m.

**Figure 8:**
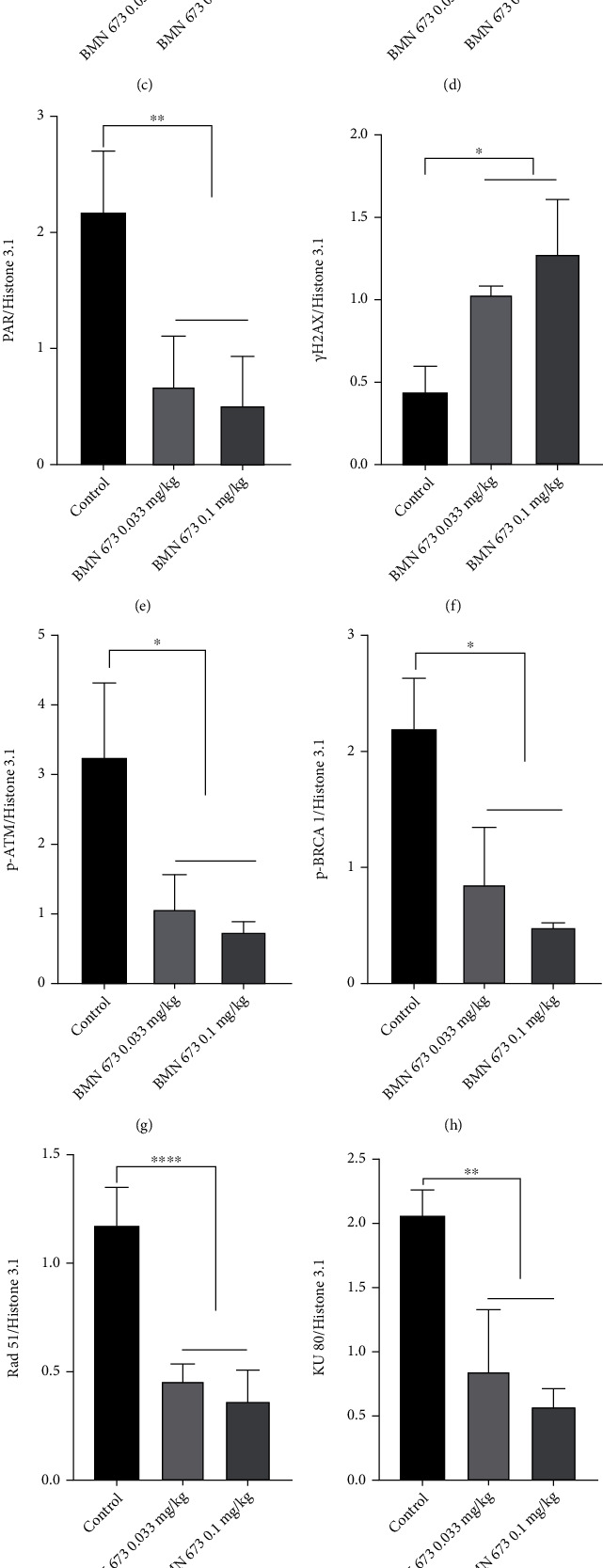
PARP1 inhibition impaired the capacity of DNA repair and activated the Cyto c/Cas 3 pathway. (a–m) Western blot showed that expression of *γ*H2AX was upregulated in the hair follicle in mice treated with BMN 673. There were no significant changes in the expression of PARP1 in mice treated with BMN 673, while the expression of PAR was downregulated. Expression of DNA damage repair proteins, p-ATM, p-BRCA 1, Rad51, KU80, and KU70, was decreased in mice treated with BMN 673. Translocation of AIF to the nucleus was blocked in the hair follicle, while expression of Cyto c was upregulated and Cas 3 was activated in mice treated with BMN 673. ^∗^*P* < 0.05, ^∗∗^*P* < 0.01, ^∗∗∗^*P* < 0.001, and ^∗∗∗∗^*P* < 0.0001.

PARP1 participates in AIF-PARP1-mediated caspase-independent apoptosis and DNA damage and repair, which is involved in hair cycles. Therefore, we treated mice with the PARP1-specific inhibitor BMN 673 to explore the effects of PARP1 in hair cycles. Compared with the control, treatment with BMN 673 at 0.033 mg/kg dosage did not cause significant changes in body weight. However, BMN 673 at 0.1 mg/kg dosage caused severe toxicity in mice, as shown by the decreased body weight (*P* < 0.001) (Figures 7(a) and 7(b)). HE staining of dorsal skin sections also showed no significant change in HF density and skin thickness, except for decreased HF diameters in mice treated with BMN 673 at 0.033 mg/kg, compared to that of controls (*P* < 0.01) (Figures 7(c)–7(f)). On the other hand, western blotting showed that treatment with 0.033 mg/kg BMN 673 downregulated the expression of 57 kDa AIF, PAR, and DNA repair-related proteins p-ATM, p-BRCA 1, Rad51, and KU70/80 but upregulated the expression of *γ*H2AX, Cyto c, and c-Cas 3 (Figure 8).

### 3.5. Inhibition of Cas 3 Activity Enhanced DNA Damage and Repair and Activated the AIF-PARP1-Mediated Apoptotic Signalling Pathway

**Figure 9:**
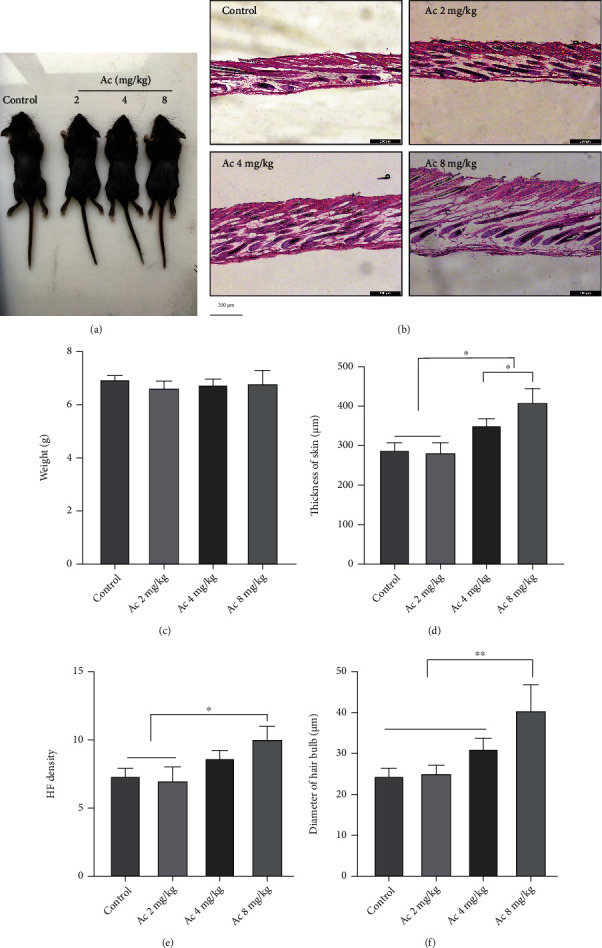
Cas 3 inhibition delayed the degeneration of hair follicles (HFs). (a) Gross observation of mice treated with Ac. (b, d) HE staining of the dorsal skin showed that skin thicknesses were increased in mice treated with 4 and 8 mg/kg Ac. (c) The weight of mice had no significant changes in mice treated with Ac. (e, f) HF density and diameters of hair bulbs were increased in mice treated with 8 mg/kg Ac. Data of HF density are presented as mean ± SD of number of HFs under 100x lens: control: 7.3 ± 0.6, Ac 2 mg/kg: 7.0 ± 1.0, Ac 4 mg/kg: 8.7 ± 0.6, and Ac 8 mg/kg: 11.0 ± 1.0. ^∗^*P* < 0.05, ^∗∗^*P* < 0.01, and ^∗∗∗^*P* < 0.001. Scale bar (b): 200 *μ*m.

**Figure 10:**
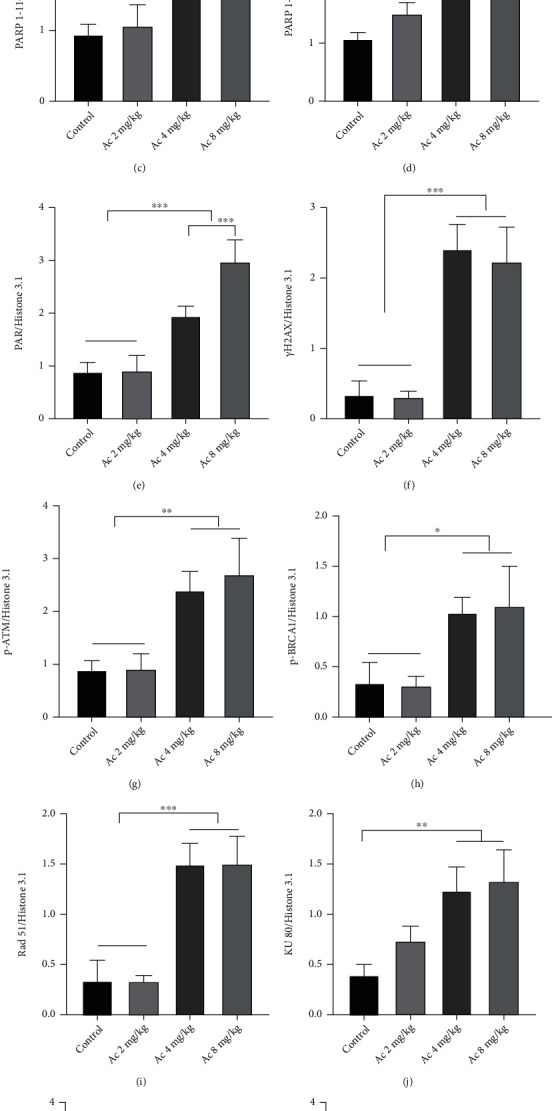
Cas 3 inhibition enhanced the capacity of DNA repair. (a–m) Western blot showed that expression of *γ*H2AX was upregulated in the hair follicle in mice treated with 4 and 8 mg/kg Ac. Expression of 116 kDa PARP1 and PAR was enhanced in mice treated with Ac as indicated. Expression of DNA damage repair proteins, p-ATM, p-BRCA 1, Rad51, KU80, and KU70, was increased in mice treated with Ac at 4 and 8 mg/kg. Translocation of AIF to the nucleus was enhanced in the hair follicle, while c-Cas 3 was downregulated in mice treated with Ac. ^∗^*P* < 0.05, ^∗∗^*P* < 0.01, and ^∗∗∗^*P* < 0.001.

As Cas 3 participates in apoptosis, DNA damage, and repair by using PARP1 as the substrate, which is involved in hair cycles, we treated the mice with the Cas 3-specific inhibitor Ac. HE staining of dorsal skin sections showed that treatment with Ac at 8 mg/kg significantly increased skin thickness, HF density, and HF diameters, compared with those in the control group (Figures 9(b) and 9(d)–9(f)). Western blotting showed that Ac treatment upregulated the expression of 57 kDa AIF, 116 kDa PARP1, and PAR, as well as DNA damage- and repair-related proteins: *γ*H2AX, p-ATM, p-BRCA 1, Rad51, and KU70/80, while downregulating c-Cas 3 expression (Figure 10). There was no significant change in the body weight (Figures 9(a) and 9(c)), indicating no Ac-related toxicity up to an 8 mg/kg dosage.

### 3.6. Seesaw Relationship Was Observed between Cyto c/Cas 3- and AIF-PARP1-Mediated Apoptosis, with PARP1 as a Possible Fulcrum

**Figure 11:**
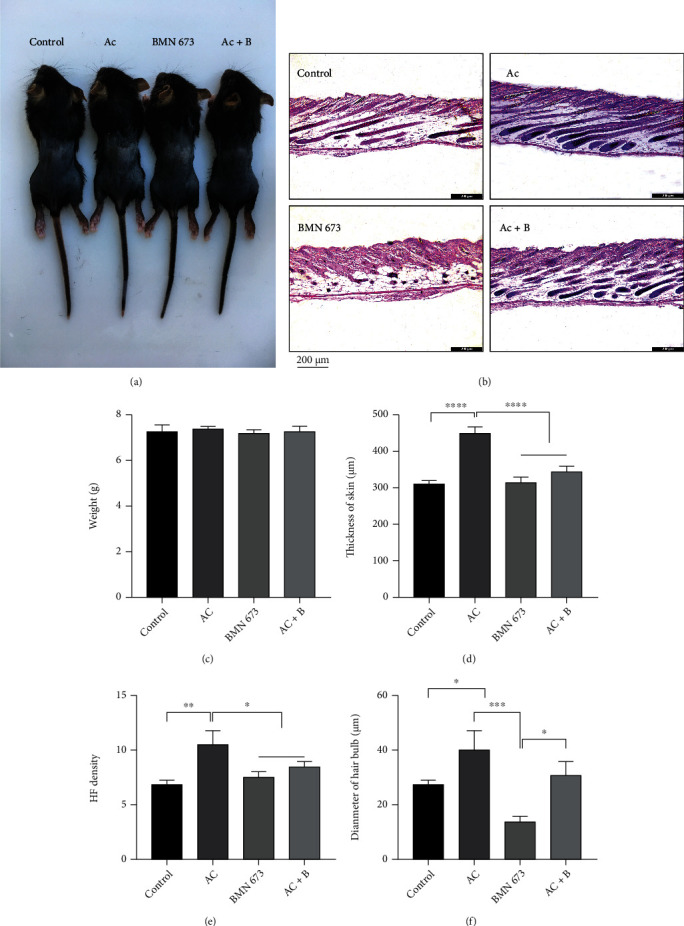
Cas 3 inhibition delayed regress of HFs, while PARP1 inhibition promoted degeneration of HFs. (a, c) There is no difference in the weight of the mice among various groups. (b, d–f) HE staining showed an increase in thickness of dorsal skin and HF density in mice treated with Ac compared to controls. Diameter of hair bulbs in mice treated with BMN 673 was decreased, while it increased in mice treated with Ac. Data of HF density are presented as mean ± SD of number of HFs under 100x lens: control: 7.3 ± 0.6, Ac: 10.7 ± 1.2, BMN 673: 7.7 ± 0.6, and Ac+B: 8.7 ± 0.6. ^∗^*P* < 0.05, ^∗∗^*P* < 0.01, ^∗∗∗^*P* < 0.001, and ^∗∗∗∗^*P* < 0.0001. Scale bar (b): 200 *μ*m.

**Figure 12:**
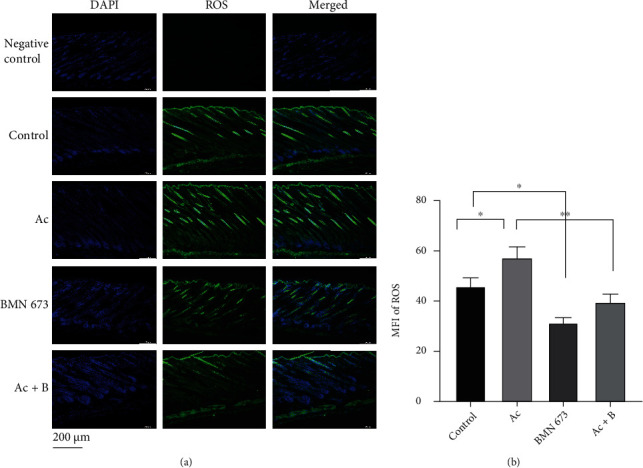
PARP1 inhibition decreased the ROS in HFs, while ROS was increased by Cas 3 inhibition. (a, b) ROS in HFs in mice treated with PARP1 or Cas 3 inhibitor was detected by probe, and the results showed that MFI of ROS in hair follicles was elevated in mice treated with Ac, while it was dropped in mice treated with BMN 673. ^∗^*P* < 0.05, ^∗∗^*P* < 0.01. Scale bar (a): 200 *μ*m.

Next, we sought to explore the temporal and spatial distribution of AIF-PARP1 and Cyto c/Cas 3 apoptotic signalling pathways and their relationship with DNA damage and repair during hair cycle transition. We treated mice with a specific PARP1 inhibitor BMN 673 (0.033 mg/kg), caspase-3 inhibitor Ac (8 mg/kg), or both and compared the effects of each treatment on AIF-PARP1- and Cyto c/Cas 3-mediated apoptotic signalling pathways and DNA damage and repair signalling pathways. Treatment with individual or combined inhibitors did not cause any significant body weight changes (Figures 11(a)and 11(c)), suggesting no toxic effects at the aforementioned dosages. However, HE staining showed that Ac treatment significantly increased skin thickness (*P* < 0.0001), HF density (*P* < 0.01), and HF diameter, compared with those in the control (*P* < 0.05) (Figures 11(b) and 11(d)–11(f)). In contrast, BMN 673 treatment significantly decreased HF diameters (*P* < 0.05) (Figures 11(b) and 11(f)). Nevertheless, combined treatment rescued the decrease in HF diameter caused by BMN 673 treatment (Figures 11(b) and 11(f)). The ROS assay showed that Ac treatment significantly increased ROS MFI (*P* < 0.05). In contrast, BMN 673 treatment significantly decreased the MFI of ROS (*P* < 0.05). However, Ac and BMN 673 combined treatment rescued the decrease in ROS MFI caused by BMN 673 treatment (*P* < 0.05) (Figures 12(a) and 12(b)).

**Figure 13:**
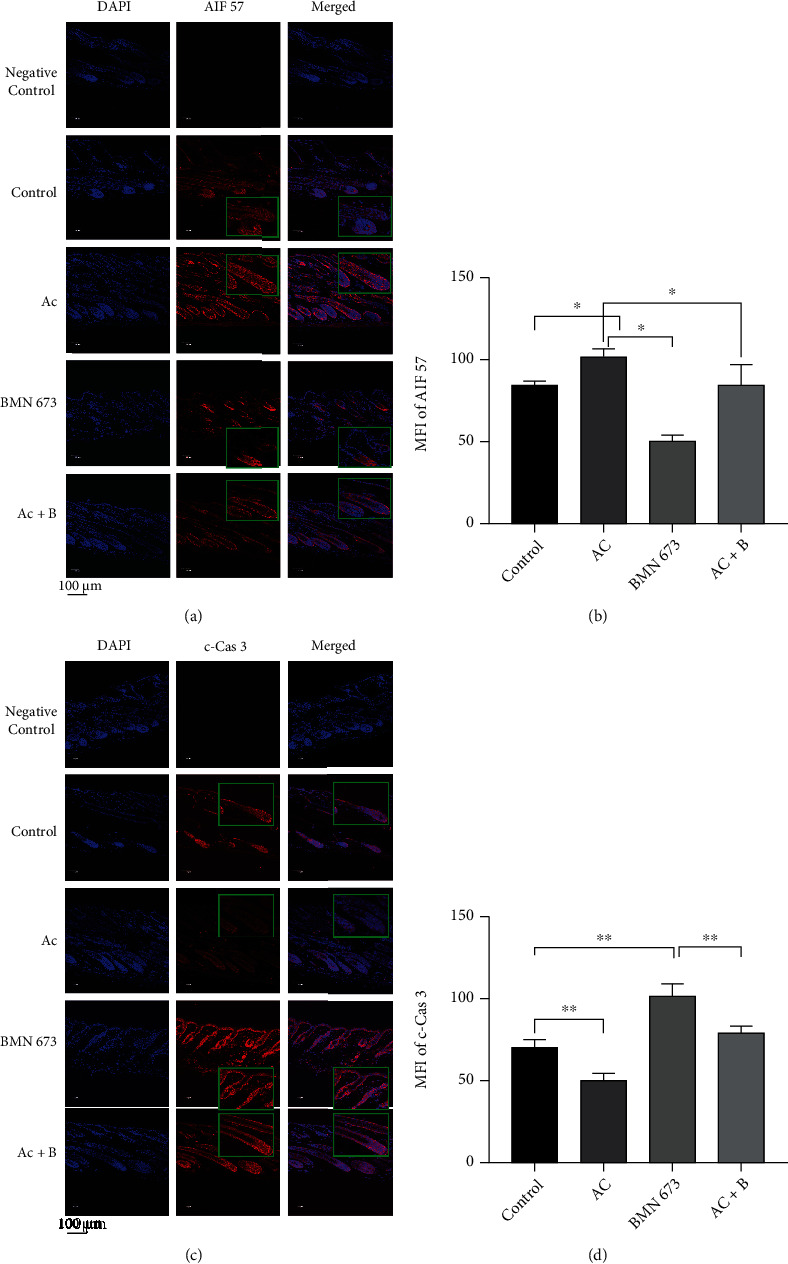
Cas 3 inhibitor reduced the activity of Cas 3 but upregulated 57 kDa AIF in HF cells, whereas the PARP1 inhibitor reduced the expression of 57 kDa AIF but promoted Cas 3 activity. (a, b) Immunofluorescence staining showed that MFI of 57 kDa AIF was increased in the hair follicle in mice treated with Ac, while it was decreased in mice treated with BMN 673. (c, d) Immunofluorescence staining showed that MFI of c-Cas 3 was decreased in the hair follicle in mice treated with Ac, while it was increased in mice treated with BMN 673. ^∗^*P* < 0.05, ^∗∗^*P* < 0.01. Scale bar (a, c): 100 *μ*m.

Further, immunofluorescence staining showed that Ac treatment significantly increased MFI of 57 kDa AIF, compared with that in the control (*P* < 0.05). In contrast, BMN 673 treatment significantly decreased the MFI of 57 kDa AIF (*P* < 0.05). However, Ac and BMN 673 combined treatment rescued the decrease in MFI of 57 kDa AIF caused by BMN 673 treatment (*P* > 0.05, control vs. Ac+B) (Figures 13(a) and 13(b)).

**Figure 14:**
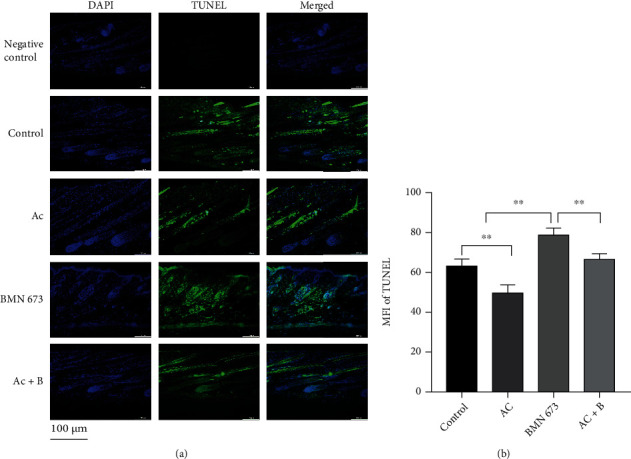
Apoptotic cells in HFs were decreased in mice treated with Ac, while they were increased in mice treated with BMN 673. (a, b) TUNEL staining showed that MFI of TUNEL in the follicles was decreased in mice treated with Ac, while it was increased in mice treated with BMN 673. ^∗∗^*P* < 0.01. Scale bar (a): 100 *μ*m.

Ac treatment decreased the MFI of c-Cas 3 (*P* < 0.01), while BMN 673 treatment significantly increased it (*P* < 0.01), and combined treatment with Ac and BMN 673 rescued the decrease in MFI of c-Cas 3 (*P* < 0.01) (Figures 13(c) and 13(d)). Similarly, TUNEL staining showed that Ac treatment decreased the MFI of tunnel staining (*P* < 0.01), while BMN 673 treatment significantly increased it (*P* < 0.01) (Figures 14(a) and 14(b)).

**Figure 15:**
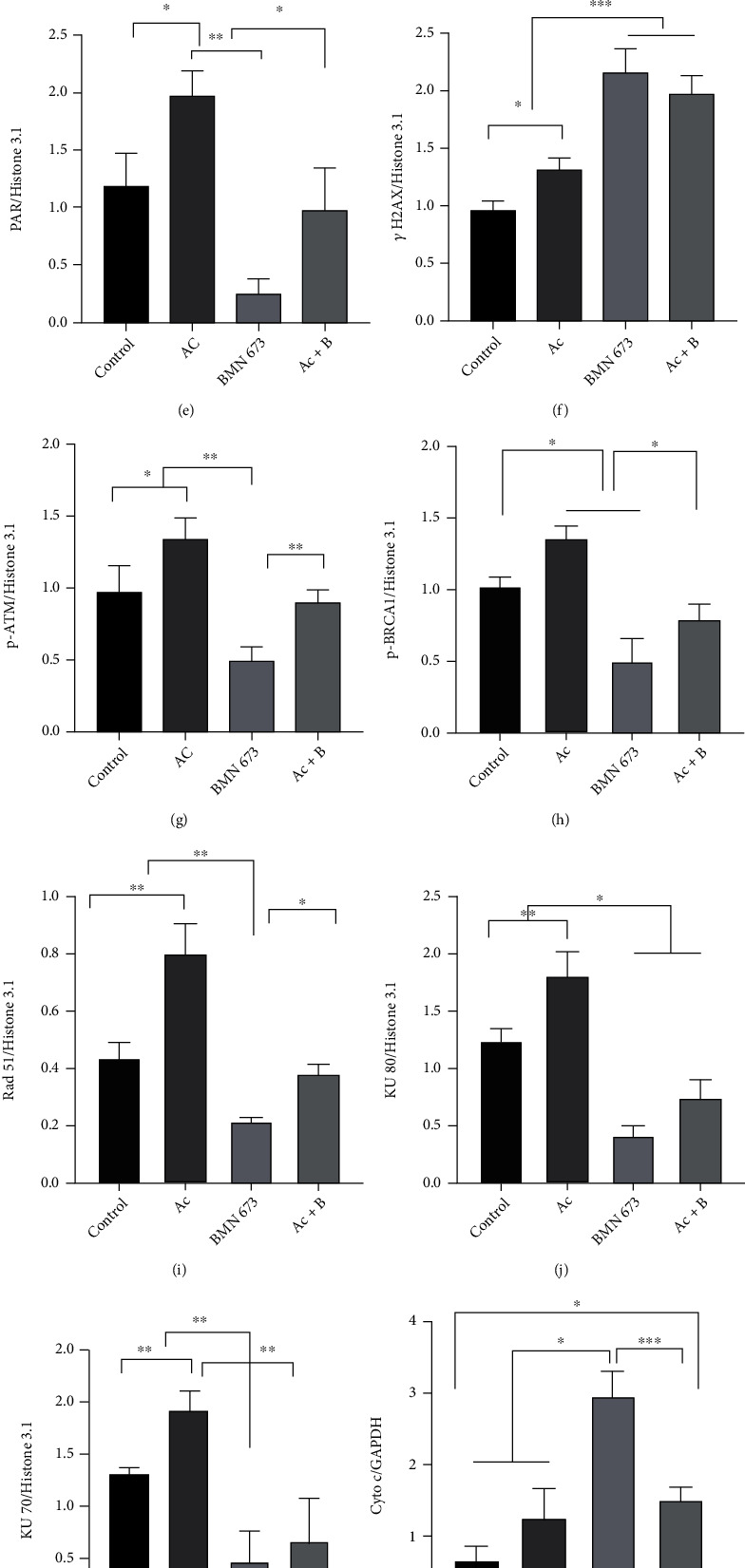
Cas 3 inhibition promoted double-strand break (DSB) repair in HF cells, and PARP1 inhibition promoted activation of the Cyto c/Cas 3 pathway. (a–m) The results of western blot showed that the marker of DNA break *γ*H2AX was upregulated in mice treated with inhibitors. Expression of 116 kDa PARP1 and PAR in the hair follicle was increased in mice treated with Ac, followed by the DNA repair proteins, while AIF translocation was enhanced and activation of Cas 3 was weakened. Expression of PAR was decreased in mice treated with BMN 673, followed by the DNA repair proteins, while expression of Cyto c and activation of Cas 3 were enhanced and AIF translocation was weakened. There was no significant change in the expressions of DNA repair proteins, except an increase in *γ*H2AX and Cyto c and decrease in KU80 in mice treated with both Ac and BMN 673 (Ac+B). (n, o) More NAD+ and NADH were consumed in hair follicles in mice treated with Ac, in contrast to the mice treated with BMN 673. ^∗^*P* < 0.05, ^∗∗^*P* < 0.01, ^∗∗∗^*P* < 0.001, and ^∗∗∗∗^*P* < 0.0001.

Western blotting showed that compared with that in the control group, Ac treatment upregulated the expression of 57 kDa AIF, 116 kDa PARP1 (*P* < 0.05), and PAR as well as DNA damage- and repair-related proteins *γ*H2AX, p-ATM, p-BRCA 1, Rad51, and KU70/80 but downregulated c-Cas 3 expression (Figures 15(a)–15(m)). However, BMN 673 treatment downregulated the expression of 57 kDa AIF, PAR, and DNA repair-related proteins p-ATM, p-BRCA 1, Rad51, and KU70/80 but upregulated the expression of *γ*H2AX, Cyto c, and c-Cas 3 (Figures 15(a)–15(m)). Compared with that in the control, combined BMN 673 and Ac treatment upregulated the expression of *γ*H2AX (*P* < 0.001) and Cyto c (*P* < 0.05) (Figures 15(a), 15(f), and 15(l)) but downregulated KU80 expression (*P* < 0.05) (Figures 15(a) and 15(j)). Interestingly, BMN 673 and Ac treatment suppressed Ac-induced upregulation of 57 kDa AIF and PAR as well as all the DNA repair-related proteins but rescued Ac-induced downregulation of c-Cas 3 (Figures 15(a)–15(m)). Once again, combined BMN 673 and Ac treatment maintained the high expression of *γ*H2AX comparable to BMN 673 treatment, higher than Ac treatment (Figures 15(a) and 15(f)). In addition, more NAD+ and NADH were consumed in hair follicles in mice treated with Ac, in contrast to the mice treated with BMN 673(Figures 15(n) and 15(o)).

### 3.7. Macrophages Were Recruited and Polarized during the Hair Cycle

**Figure 16:**
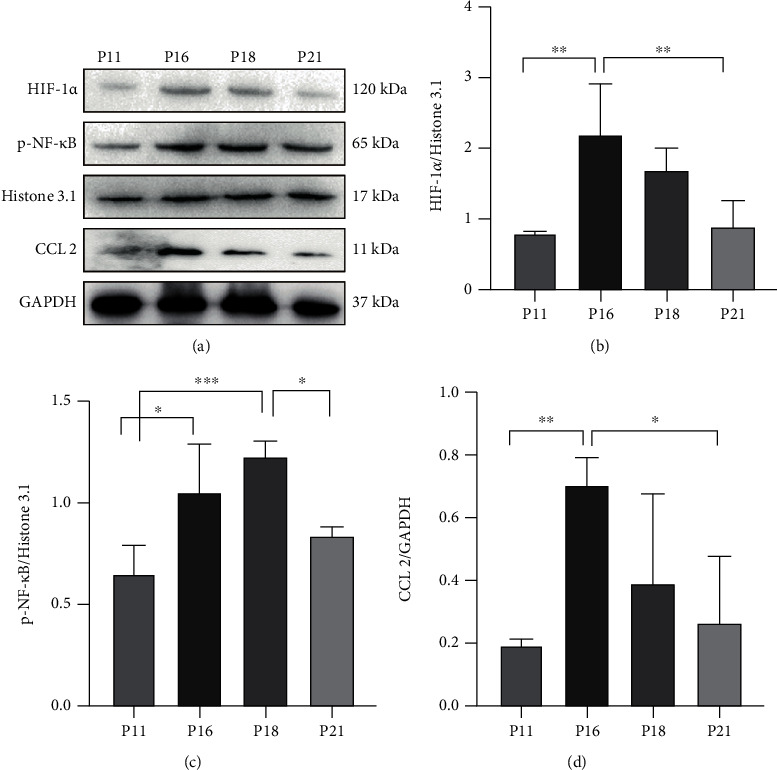
Expressions of the proteins for macrophage recruitment and polarization were changed during the hair cycle. (a, b) Expression of HIF-1*α* in the nucleus was upregulated significantly in catagen. (a, c, d) p-NF-*κ*B in the nucleus was upregulated in catagen, and the expression of CCL2 was increased significantly in early catagen. ^∗^*P* < 0.05, ^∗∗^*P* < 0.01, and ^∗∗∗^*P* < 0.001.

**Figure 17:**
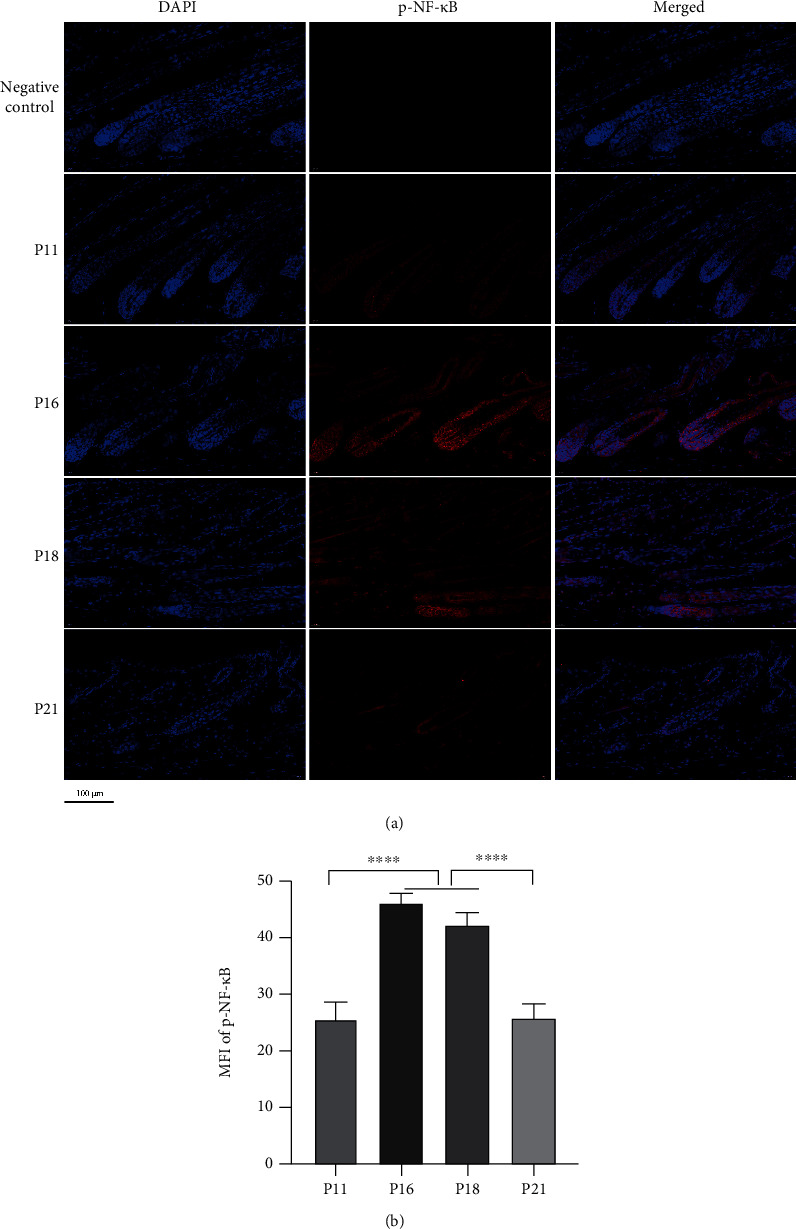
NF-*κ*B was activated during the hair cycle. (a, b) Immunofluorescence staining showed that p-NF-*κ*B was expressed widely in skin and hair follicles, and MFI of p-NF-*κ*B increased in catagen. ^∗∗∗∗^*P* < 0.0001. Scale bar (a): 100 *μ*m.

**Figure 18:**
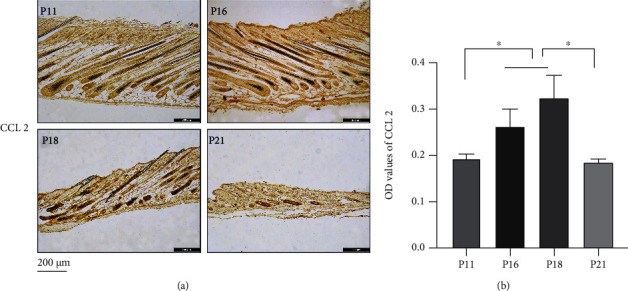
CCL2 was upregulated in catagen. (a, b) Immunohistochemical staining showed that CCL2 was expressed widely in skin and hair follicles, and it was upregulated in catagen. ^∗^*P* < 0.05. Scale bar (a): 200 *μ*m.

**Figure 19:**
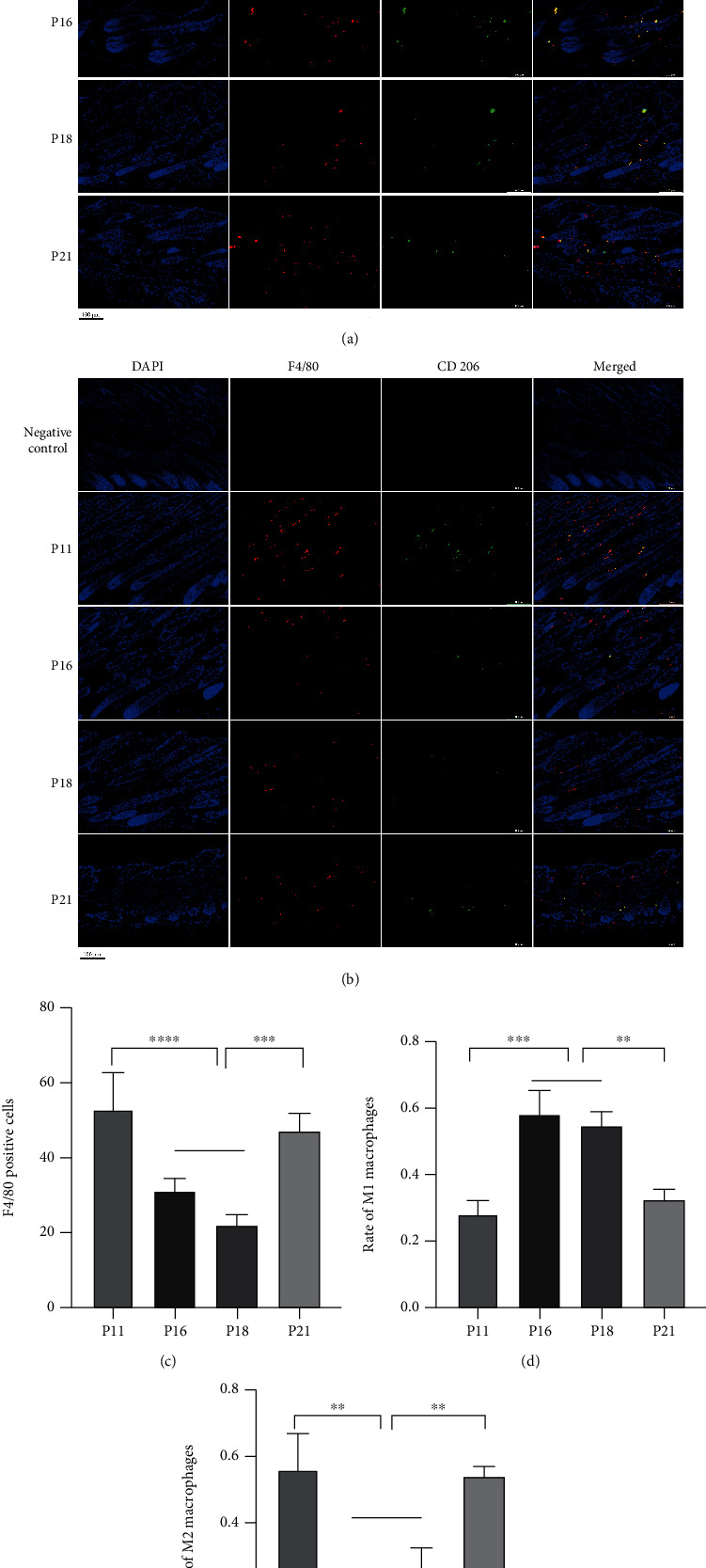
Macrophages were recruited and polarized in the hair cycle. (a–c) F4/80-positive cells (macrophage) were distributed in the epidermis and around HFs, and the number decreased significantly in catagen and recovered in telogen. (a, d) F4/80^+^CD86^+^ cells (M1 macrophage) were scattered around HFs, and the rate increased significantly in catagen. (b, e) F4/80^+^CD206^+^ cells (M2 macrophage) were distributed around HFs, and the rate was reduced significantly in catagen, while it was recovered in telogen. ^∗∗^*P* < 0.01, ^∗∗∗^*P* < 0.001, and ^∗∗∗∗^*P* < 0.0001. Scale bar (a, b): 100 *μ*m.

To investigate the distribution and polarization of macrophages during the hair cycle, the surface markers of macrophages, p-NF-*κ*B and CCL2, were analysed via immunohistochemistry and western blotting. Results showed that p-NF-*κ*B and CCL2 were expressed during the whole cycle and were upregulated significantly from anagen to early catagen (*P* < 0.05) (Figures 16(a), 16(c), and 16(d)). In addition, expression of HIF-1*α* was upregulated significantly from anagen to catagen (Figures 16(a) and 16(b)). Immunofluorescence analysis showed that p-NF-*κ*B was distributed in the HFs and interstitium, while the signal intensity was enhanced significantly in catagen compared with anagen (*P* < 0.0001) (Figures 17(a) and 17(b)). Downstream, CCL2 showed similar expression trends (*P* < 0.05) (Figures 18(a) and 18(b)). Macrophages were distributed abundantly in the epidermis and around HFs in anagen and significantly decreased from anagen to catagen (*P* < 0.0001); however, the number was restored significantly in telogen (*P* < 0.001) (Figures 19(a)–19(c)). For the polarization of macrophages during the hair cycle, the results showed that M1 macrophages marked by F4/80 and CD86 rarely existed in the epidermis and around HFs, but an increase in the rate of M1 macrophages was observed from anagen to catagen, and there were M1 macrophages infiltrating around the HFs in telogen (Figures 19(a) and 19(d)). M2 macrophages marked by F4/80 and CD206 infiltrated abundantly in the epidermis and around HFs in anagen, and the rate of M2 macrophages decreased rapidly from anagen to catagen; however, the rate was recovered in telogen (Figures 19(b) and 19(e)).

## 4. Discussion

In this study, we found that intrinsic ROS in hair follicles was increased in the catagen phase followed by increased DNA breaks and DNA repair while the capacity of DNA repair was finite, which resulted in the activation of AIF-PARP1 and Cyto c/Cas 3 apoptotic pathways. Meanwhile, macrophages were polarized to M1 or M2 type at different stages of the hair cycle.

In anagen, the hair papilla exchanges information with the hair bulge, and the hair follicle cells proliferate frequently, which results in the elongation of HFs [30]. Oxidative respiration provides power to the growth of HFs. ROS are mainly produced during cell respiration [31]. ROS can be used as a second messenger to transmit signals in cells and play an important role in cell proliferation. It was reported that intrinsic ROS are involved in the regulation of the hair cycle. ROS in small amounts activates the proliferation of hair follicle stem cells and promotes the hair cycle in anagen by promoting transient Src kinase phosphorylation and activation of the prolactin family 2 subfamily c of growth factors or regulating VEGF and GDNF expression through the MAPK/ERK pathway [6, 32]. In this study, ROS in HFs remained at low levels, and numerous Ki67-positive cells gathered at the matrix area in the anagen phase, which was consistent with the results of previous studies.

In catagen, the proliferation of hair follicle cells slowed down and even stagnated, and apoptosis took the upper hand followed by the degeneration of HFs, which may be related to ROS accumulation. It has been previously reported that the increase in ROS is a key factor in hair follicle degeneration during catagen [8] which mostly results from oxidative damage, but the specific mechanism remains unclear. Excessive ROS can cause oxidative damage to biological macromolecules in cells and is related to DNA damage, which leads to apoptosis [33, 34]. In this study, during the degenerative phase of HFs, the level of ROS was significantly increased, while DNA damage in hair follicle cells was also significantly increased. To maintain genome integrity and cell survival, cells activate DNA repair mechanisms to deal with DNA breaks [35]. DNA breaks induced by ROS can be repaired through BER, HR, or NHEJ pathways. PARP1 is necessary to guide these pathways [36–38] by recognizing DNA breaks and using NAD+ as a substrate and participates in DNA repair [39, 40], to maintain DNA integrity and stability. In early catagen, with the increase in ROS and DNA breaks, PARP1 and the key proteins of HR and NHEJ repair pathways were significantly upregulated compared to those in anagen, while the BER pathway repair protein OGG1 was significantly downregulated. This indicated that hair follicle cells quickly activate HR and NHEJ repair pathways and preferentially repair the double-strand breaks (DSBs), which have more serious consequences, to save themselves. Expression of PARP1 and activity of ATM were upregulated when DSBs, which were marked by *γ*H2AX, increased. BRCA 1 is recruited and activated to carry Rad51 to DSBs and repair the breaks in the HR pathway. In addition, KU70 and KU80 can be recruited to repair DSBs in the NHEJ pathway. However, the capacity of cells to repair DNA damage is limited. In the late stage of catagen, when DNA damage was still present in large quantities, the DNA damage repair proteins in hair follicle cells were significantly downregulated, which indicates that the hair follicle cells fail to save themselves in the degenerative stage. According to the principles of DNA repair, HR is an accurate DNA repair method, and it is possible that some hair follicle cells survive as seed cells to enter the next cycle through the HR pathway. While cells repaired by the NHEJ method often have genetic mutations, to maintain the integrity of the genome and prevent the occurrence of tumours, these cells may undergo apoptosis similar to those that have not been repaired. In catagen, after DNA damage, most hair follicle cells undergo apoptosis. Apoptosis of hair follicle cells is related to the activation of the AIF-PARP1 pathway and the Cyto c/Cas 3 pathway. It was found that more AIF translocated to the nucleus in catagen than in anagen and telogen, and the concentration of NAD+ and NADH in hair follicle cells decreased rapidly. The increase in 89 kDa PARP1 and PAR induced an increase in AIF translocation from anagen to catagen, which resulted in the apoptosis of hair follicle cells. The Cyto c/Cas 3 apoptotic pathway is also involved in the apoptosis of hair follicle cells, but what is puzzling is that the expressions of Cyto c and c-Cas 3 were lower in the early stage of catagen. To confirm the roles of apoptosis mediated by PARP1 or Cas 3 in the hair cycle, mice were treated with the PARP1 inhibitor and Cas 3 inhibitor. Cas 3 inhibition delayed the degeneration of HFs, and apoptosis of hair follicle cells was weakened, which may be related to the increase in PARP1 activity and the promotion of DNA repair. A previous study showed that Cas 3 is required to inhibit electron transport through the electron transfer chain and reduce ROS production, while Cas 3 deficiency resulted in an increase in ROS [41]. Similarly, generation of ROS in HFs was increased in mice treated with the Cas 3 inhibitor in this study. However, the inhibition of Cas 3 increased the nuclear translocation of AIF in hair follicle cells. In addition, expression of 89 kDa PARP1 was increased, along with the upregulation with 116 kDa PARP1, in Cas 3-inhibited mice, indicating that another form of PARP1 cleavage may exist, other than Cas 3, in hair follicles. Increased 89 kDa PARP1 and PAR possibly resulted in the increase in AIF translocation in mice treated with the Cas 3 inhibitor. According to the results of previous studies, PARP1 inhibition reduced ROS production by inhibiting MAPK activity [42], and analogously, ROS production was reduced in HFs in mice treated with the PARP1 inhibitor. Although the PARP1 inhibitor reduced the translocation of AIF in hair follicle cells, it inhibited the ability of DNA repair and promoted the activation of the Cyto c/Cas 3 pathway, which resulted in the increase in apoptotic cells in hair follicles. In summary, both AIF-PARP1 and Cyto c/Cas 3 apoptotic pathways were involved in mediating apoptosis of hair follicle cells, and they influenced each other, similar to the ends of a seesaw, which may be a regulatory mechanism for the body to maintain homeostasis. In addition, it is feasible to delay hair follicle regression by blocking the apoptosis pathways, but it should not impair the ability of hair follicle cells to repair DNA damage.

Macrophages participate in tissue development and construction [43]. This study showed that macrophages were widely distributed around HFs and more were polarized to the M2 type in anagen. Studies have shown that the M2 polarization of macrophages depends on the presence of low levels of ROS, and M2-type macrophages are involved in secreting growth factors (such as VEGF) to promote cell proliferation and tissue development [44]. Chu et al. [45] also reported that M2 macrophages secrete HGF and IGF-1 and activate the Wnt/*β*-catenin signalling pathway, which activates stem cells and facilitates hair regeneration. Thus, the existence of numerous M2 macrophages during anagen provides a biological basis for the proliferation of hair follicle cells and the development of HFs.

In our study, consistent with the results of Hardman et al. [27], the total number of macrophages decreased in catagen, which might be attributed to apoptosis. We found that the level of ROS in the degenerative phase of hair follicle was significantly elevated; meanwhile, the expression of HIF-1*α* and the rate of M1 macrophages were increased. In agreement with the study from the early group, increasing ROS upregulated the expression of HIF-1*α* in macrophages and promotes the polarization of macrophages towards the M1 type [46]. It was also reported that elevation of ROS increased the stability of HIF-1*α*, and HIF-1*α* participated in mediating the polarization of macrophages towards the M1 type [47]. Conversely, inhibition of HIF-1*α* results in a polarization disorder in M1-type macrophages [48], which indicates that HIF-1*α* is an important factor in promoting the polarization of macrophages to the M1 type. Accompanied by upregulation of HIF-1*α*, production of CCL2 was significantly upregulated in catagen, which may contribute to the induction of macrophage polarization towards the M1 type. It was reported that CCL2 is the mediator in the process of HIF-1*α* resulting in M1-type polarization of macrophages [49]. The upregulation of ROS and PARP1 promotes the activation of NF-*κ*B, and consequently, as the downstream of NF-*κ*B, CCL2 is activated, which promotes the recruitment and polarization of macrophages to the M1 type [50–52]. It was reported that macrophages produce large amount of ROS through the “oxidative burst” process, which further promotes tissue damage such as nervous system damage [53, 54]. This suggests that ROS generated by macrophages may in turn cause damage to hair follicles and accelerate the process of the hair cycle. Except for the factors which lead to alterations in the polarization of macrophages, during the progress of clearance of apoptotic cells, dying cells transmit signals that attract macrophages [21]. It was suggested that apoptotic cells can promote macrophage polarization to the M1 type [43], and M1 macrophages plays an important role in promoting the death and being identified of dying cells [55]. To maintain homeostasis, apoptotic cells should be eliminated and can be cleared by macrophages without inflammation, in a process referred to as efferocytosis that is inseparable from the polarization of M2-type macrophages [56]. Therefore, to maintain homeostasis, increased M2 macrophages in telogen removed the large numbers of apoptotic hair follicle cells. In addition, M2 macrophages can secrete growth factors such as HGF and IGF-1 and activate the Wnt/*β*-catenin signalling pathway; therefore, the increase in M2 macrophages possibly activates the proliferation of seed cells that survive DNA breaks and promotes the development of a new anagen.

This study has some limitations. First, the concentration threshold of ROS at the transition between promoting cell proliferation and causing apoptosis in HFs cannot be determined. In addition, the PARP1 inhibitor was selected to inhibit the translocation of AIF, and inevitably, the DNA repair effects of PARP1 were inhibited. Better methods for ROS detection and better inhibitors are warranted.

## 5. Conclusions

This study reported that the level of intrinsic ROS in hair follicles was elevated, and apoptosis of HF cells was enhanced from the anagen to catagen phase. AIF-PARP1 and Cyto c/Cas 3 pathways worked together to induce apoptosis of HF cells, like two ends of a seesaw. DNA breaks in HF cells were increased from anagen to catagen, and the capacity of DNA repair guided by HR and NHEJ was enhanced during the transition from anagen to early catagen. Macrophages are also involved in degeneration and regeneration. More M1 macrophages appeared in catagen than in anagen, which may participate in promoting the apoptosis and clearance of HFs, while more M2 macrophages were found in anagen and telogen than in catagen, which may participate in the regeneration and growth of HFs.

## Data Availability

The data used to support the findings of this study are available from the corresponding author upon request.

## Supplementary Materials

Supplementary 1 Table S1: antibodies used in western blot.

Supplementary 2 Table S2: antibodies used in immunohistochemistry and immunofluorescence staining.

Supplementary 3 Figure S1: HE staining of skin at different stages of the hair cycle. Figure S2: Ki67 and TUNEL costaining of skin at different stages of the hair cycle. Figure S3: AIF-57 and TUNEL costaining of skin at different stages of the hair cycle (enlargement of the inset images in Figure 2(a)).
