# Supplementary material for: Intrinsic ROS Drive Hair Follicle Cycle Progression by Modulating DNA Damage and Repair and Subsequently Hair Follicle Apoptosis and Macrophage Polarization
Source: Oxid Med Cell Longev. 2022 Jul 14;2022:8279269. doi: 10.1155/2022/8279269 (PMC9315455; doi:10.1155/2022/8279269)
Supplement: Supplementary 1 — Table S1: antibodies used in western blot. [file 8279269.f1.pdf]

Table S1

Primary antibodies used for Western blot.

Antibodies	Dilution	Brand	Country
γH2AX	1:1000	Abcam	US
OGG1	1:1000	Gene Tex	US
PARP 1	1:1000	Santa Cruz	US
PAR	1:1000	CST	US
p-ATM	1:1000	Merck	Germany
KU70	1:1000	Gene Tex	US
KU80	1:2000	Abcam	US
p-BRCA 1	1:1000	Abcam	US
Rad 51	1:1000	Gene Tex	US
AIF 67	1:1000	CST	US
AIF 57	1:500	Santa Cruz	US
Cleaved-Caspase 3	1:1000	CST	US
Cytochrome c	1:1000	CST	US
COX IV	1:1000	CST	US
HIF-1α	1:1000	CST	US
p-NF-KB	1:500	ABclonal	China
CCL2	1:500	ABclonal	China
Histone 3.1	1:5000	Sanjian	China
β-actin	1:5000	Sanjian	China
GAPDH	1:10000	Proteintech	China
