# Supplementary material for: Intrinsic ROS Drive Hair Follicle Cycle Progression by Modulating DNA Damage and Repair and Subsequently Hair Follicle Apoptosis and Macrophage Polarization
Source: Oxid Med Cell Longev. 2022 Jul 14;2022:8279269. doi: 10.1155/2022/8279269 (PMC9315455; doi:10.1155/2022/8279269)
Supplement: Supplementary 2 — Table S2: antibodies used in immunohistochemistry and immunofluorescence staining. [file 8279269.f2.pdf]

Table S2

Antibodies used for Immunohistochemistry and Immunofluorescenc

Antibodies	Dilution	Brand
KU 70	1:100	Gene Tex
OGG1	1:100	Gene Tex
cleaved Caspase 3	1:200	CST
F 4/80	1:100	CST
CD 86	1:300	CST
CD 206	1:20000	Abcam
p-NF-κB	1:200	ABclonal
CCL 2	1:200	ABclonal
AIF 57	1:50	Santa Cruz
Ki 67	1:200	Abcam
γH2AX	1:50	Abcam
8-OH-dG	1:100	Abcam
Anti-rabbit IgG (Alexa Fluor 594 conjugate)	1:1000	CST
Anti-rabbit IgG (Alexa Fluor 488 Conjugate)	1:1000	CST
Anti-mouse IgG (Alexa Fluor 594 conjugate)	1:1000	CST
Anti-mouse IgG (Alexa Fluor 488 Conjugate)	1:1000	CST
Anti-rat IgG (Alexa Fluor 555 Conjugate)	1:1000	CST

e staining.

Country
US
US
US
US
US
US
China
China
US
US
US
US
US
US
US
US
US
