# Supplementary material for: Intrinsic ROS Drive Hair Follicle Cycle Progression by Modulating DNA Damage and Repair and Subsequently Hair Follicle Apoptosis and Macrophage Polarization
Source: Oxid Med Cell Longev. 2022 Jul 14;2022:8279269. doi: 10.1155/2022/8279269 (PMC9315455; doi:10.1155/2022/8279269)
Supplement: Supplementary 3 — Figure S1: HE staining of skin at different stages of the hair cycle. Figure S2: Ki67 and TUNEL costaining of skin at different stages of the hair cycle. Figure S3: AIF-57 and TUNEL costaining of skin at different stages of the hair cycle (enlargement of the inset images in Figure 2(a)). [file 8279269.f3.zip › description of Supplementary figures.docx]

**
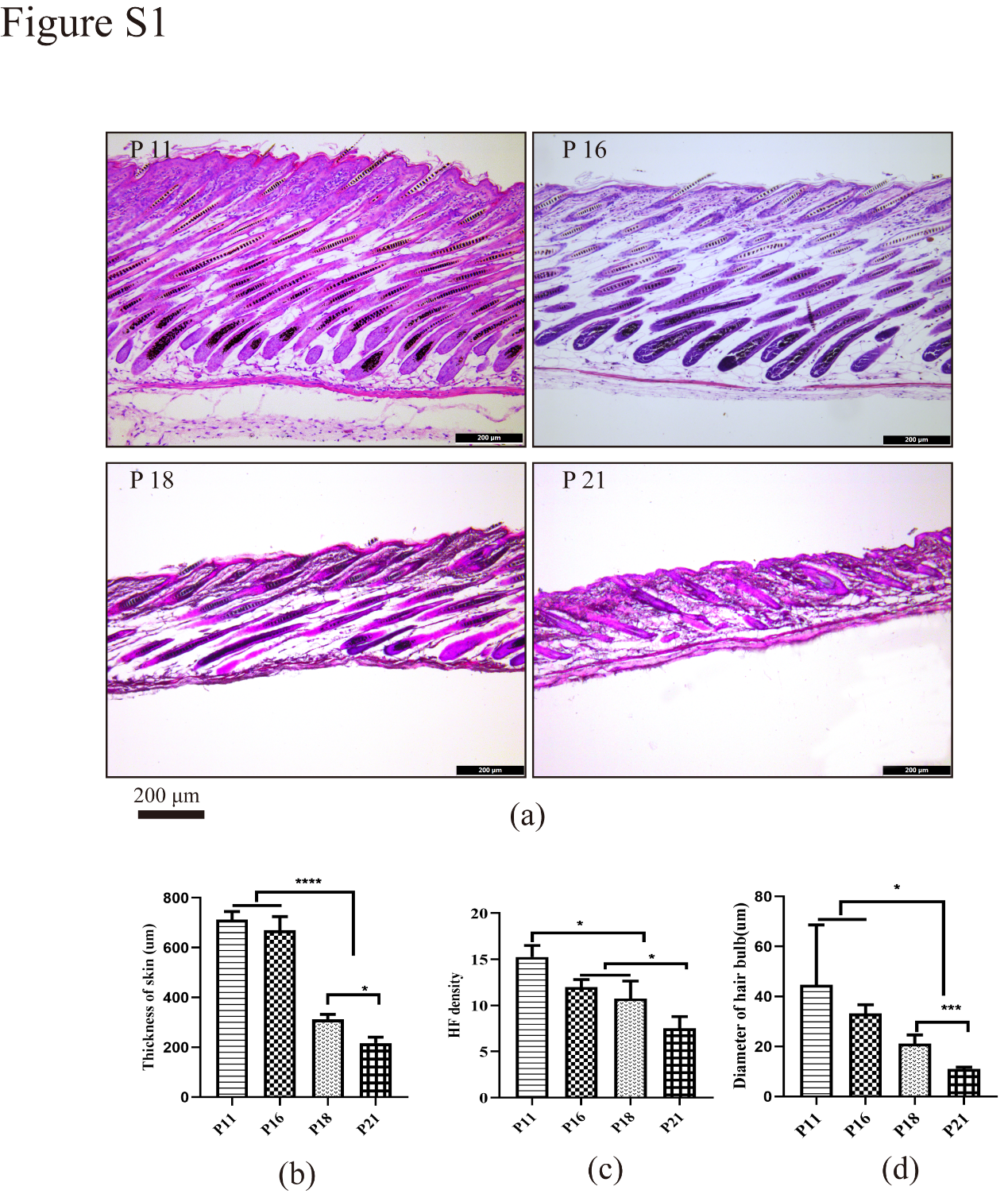
**

Figure S1. Hair follicles (HF) degenerated and dorsal skin thinned in catagen in mice. (a) HE staining of dorsal skin from postnatal day 11 (P11, anagen), P16 (early catagen), P18 (late catagen) and P21 (telogen) mice. (b) skin thickness was decreased in late catagen and telogen. (c) Hair density was decreased in catagen and telogen and data are presented as mean ± SD of number of HFs under 100x lens, P11: 15.3 ±1.2, P16: 12 ±0.8, P18: 10.7 ±1.9, P21: 7.5 ±1.3. (d) Diameters of hair bulbs were decreased in late catagen and telogen. * *P* <0.05, ** *P* <0.01, *** *P* <0.001. Scale bar (a): 200 μm.

**
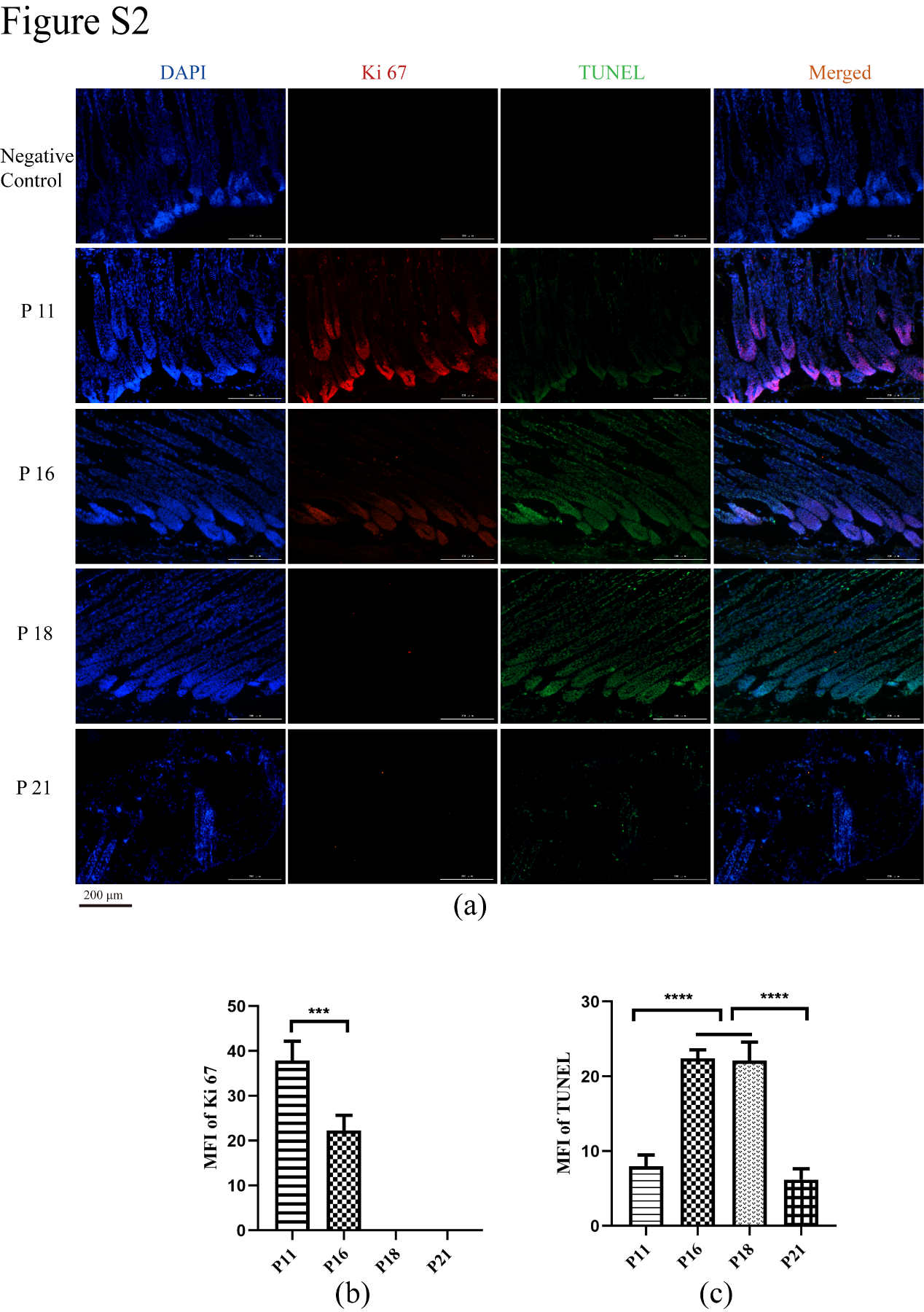
**

Figure S2. Cell proliferation decreased and apoptosis increased during the transition from anagen to catagen. (a) Proliferating cells in HFs were located in DP and matrix area in anagen and early catagen, while apoptotic cells first appeared in matrix area in anagen and gradually extend upward in catagen. (b, c) MFI of Ki 67 was decreased in early catagen, while MFI of TUNEL was elevated during anagen to catagen transition. *** *P* < 0.001, **** *P* < 0.0001. Scale bar (a): 200 μm.

**
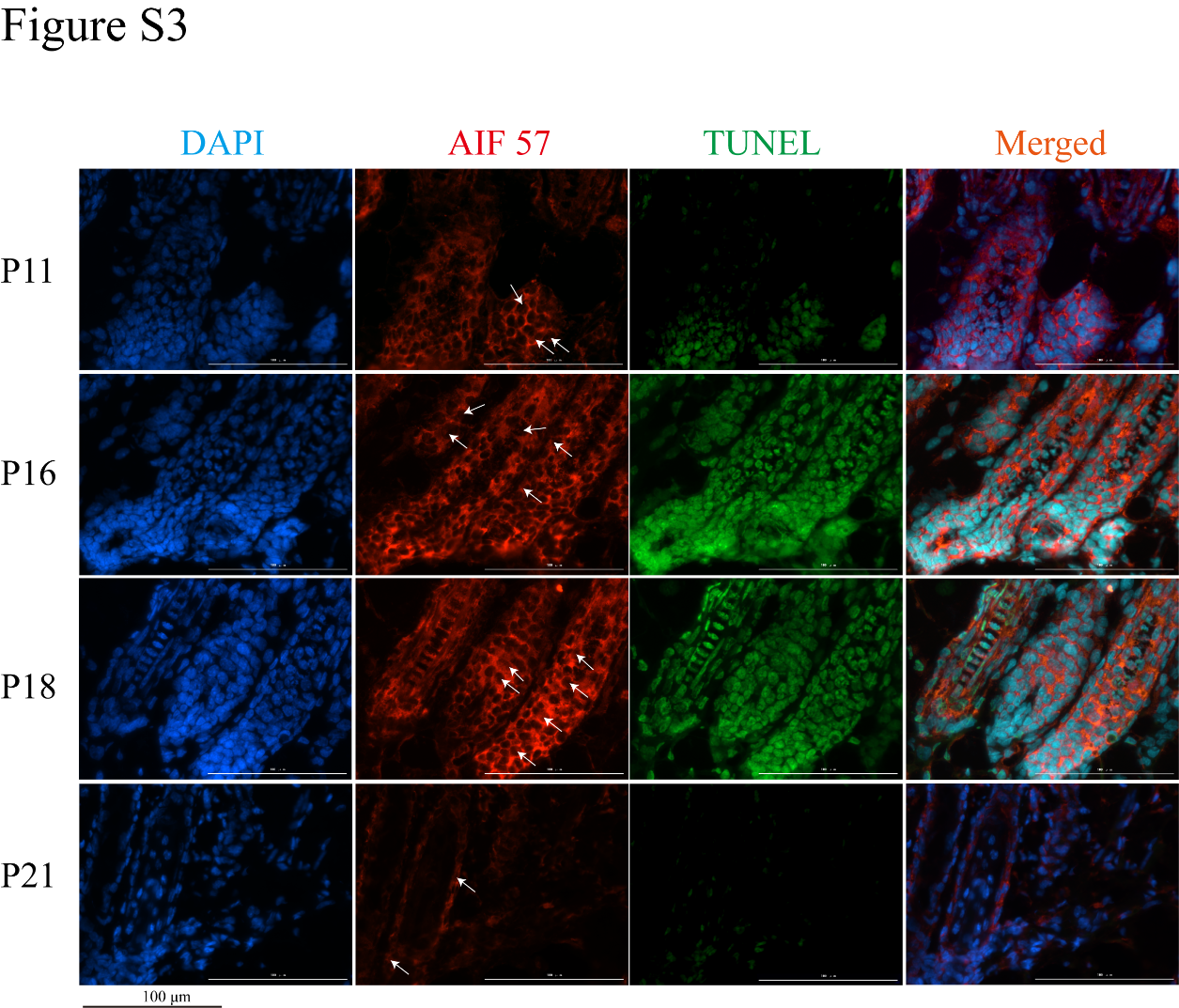
**

Figure. S3. AIF was translocated to nuclei in hair follicle cells during hair cycle. The cells with AIF translocation was marked with arrows.
