# Supplementary figures and images for: Intrinsic ROS Drive Hair Follicle Cycle Progression by Modulating DNA Damage and Repair and Subsequently Hair Follicle Apoptosis and Macrophage Polarization
Source: Oxid Med Cell Longev. 2022 Jul 14;2022:8279269. doi: 10.1155/2022/8279269 (PMC9315455; doi:10.1155/2022/8279269)

Figure S1

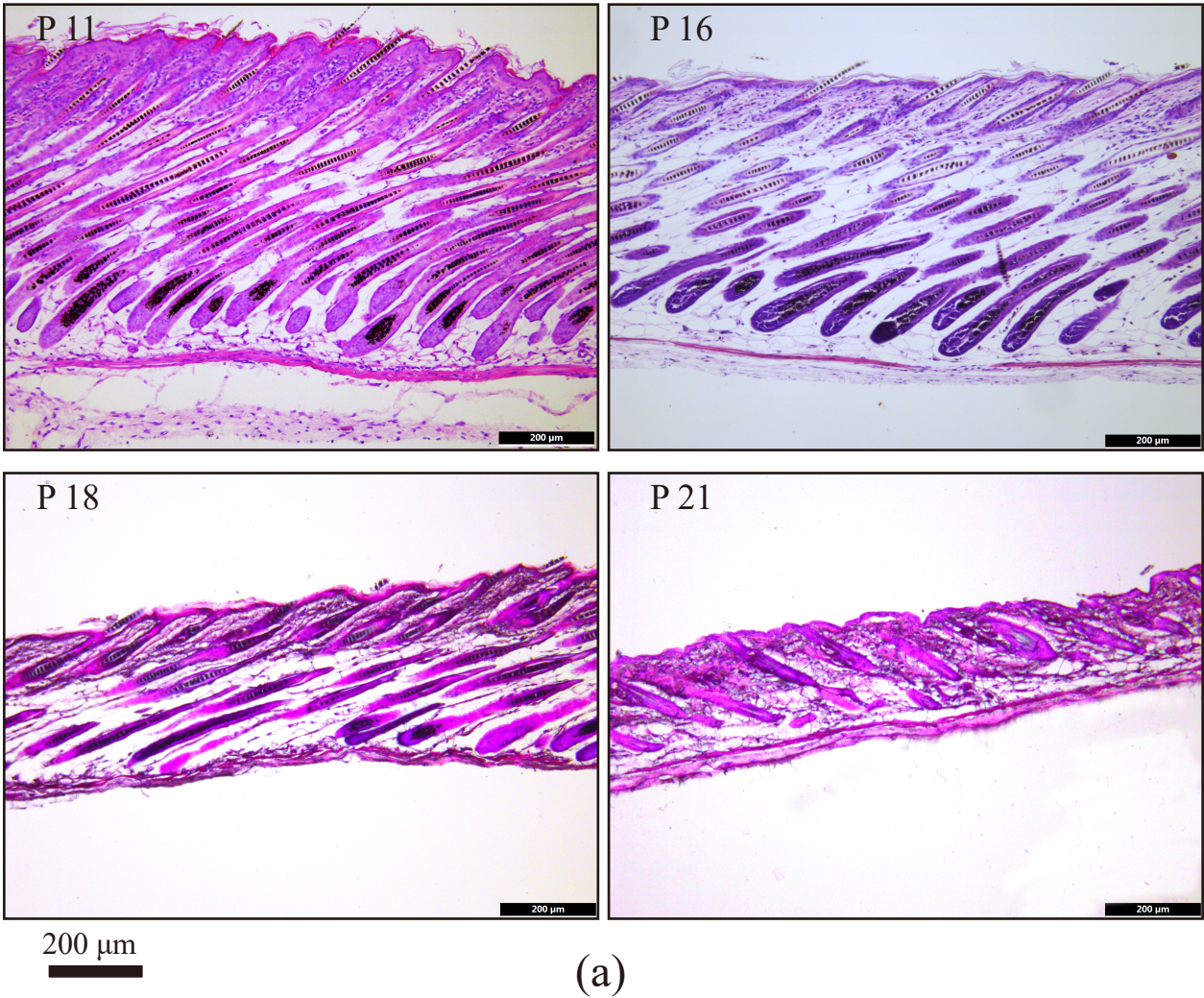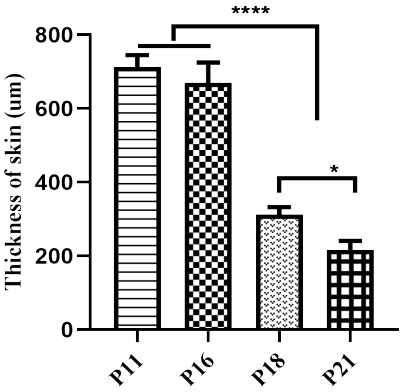

(b)

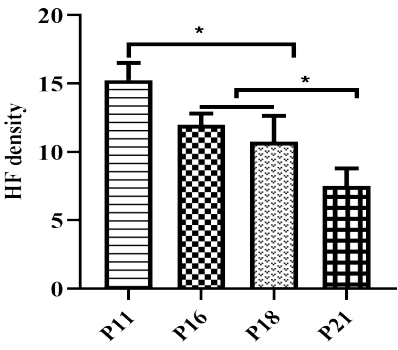

(c)

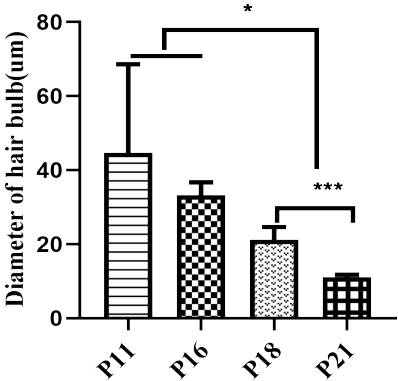

(d)

Supplement: Supplementary 3 — Figure S1: HE staining of skin at different stages of the hair cycle. Figure S2: Ki67 and TUNEL costaining of skin at different stages of the hair cycle. Figure S3: AIF-57 and TUNEL costaining of skin at different stages of the hair cycle (enlargement of the inset images in Figure 2(a)). [file 8279269.f3.zip › Figure.s1.pdf]

Figure S2

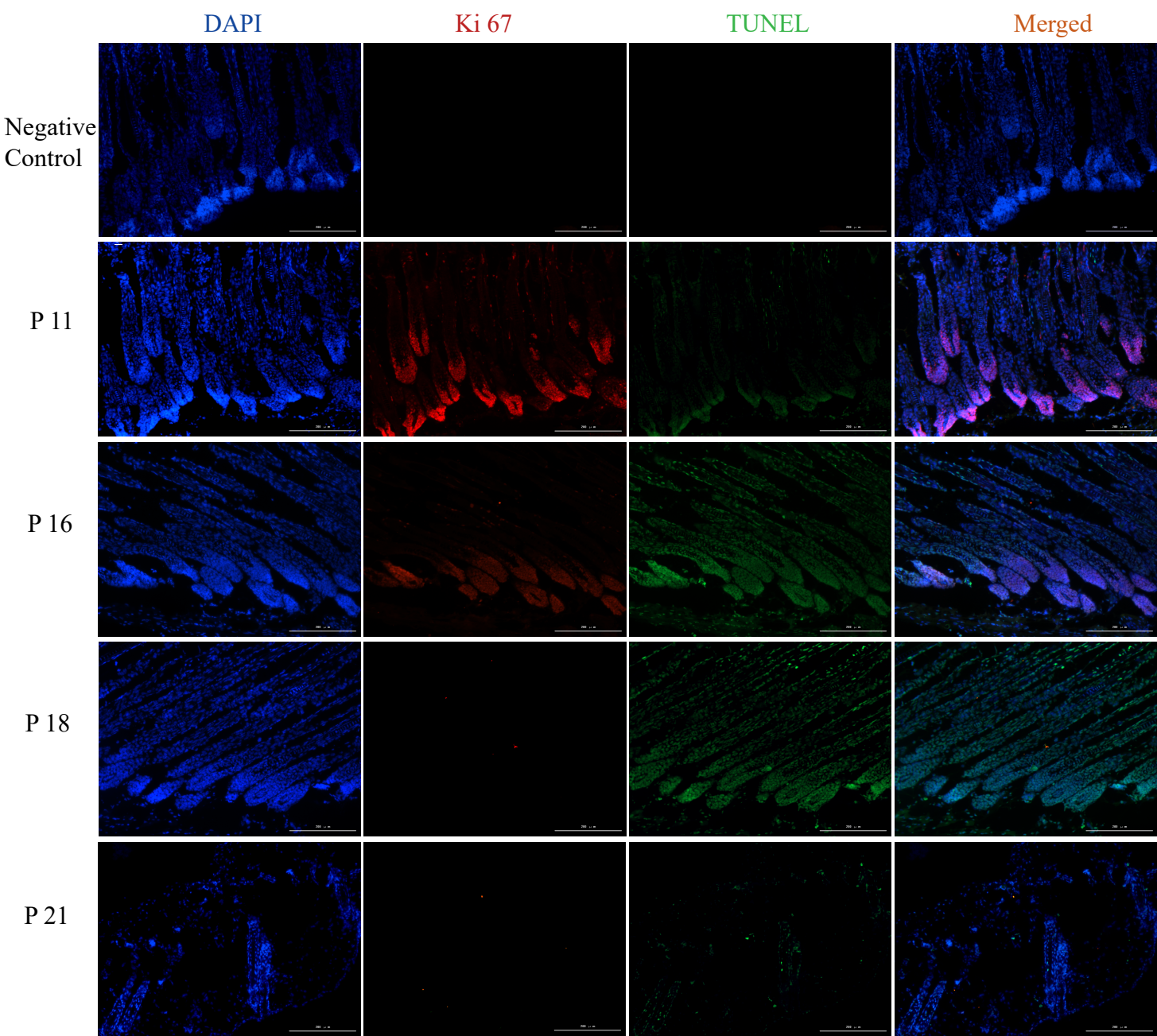

(a)

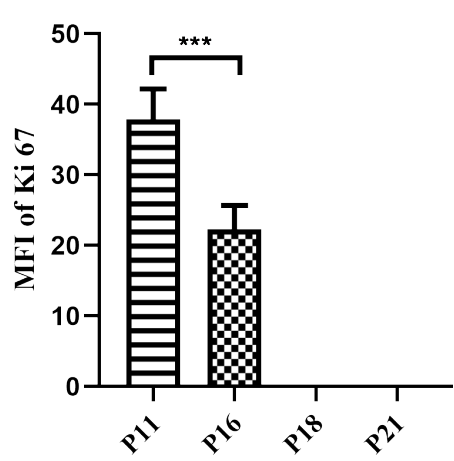

(b)

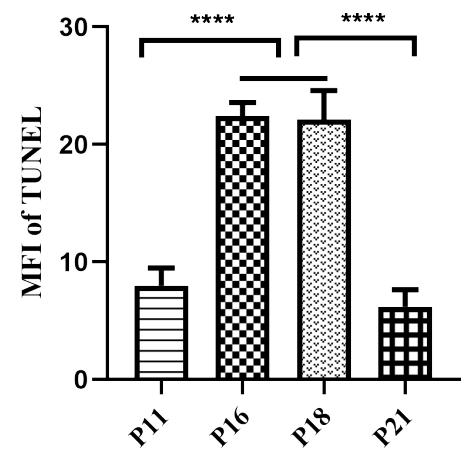

(c)

Supplement: Supplementary 3 — Figure S1: HE staining of skin at different stages of the hair cycle. Figure S2: Ki67 and TUNEL costaining of skin at different stages of the hair cycle. Figure S3: AIF-57 and TUNEL costaining of skin at different stages of the hair cycle (enlargement of the inset images in Figure 2(a)). [file 8279269.f3.zip › Figure.s2.pdf]

Figure S3

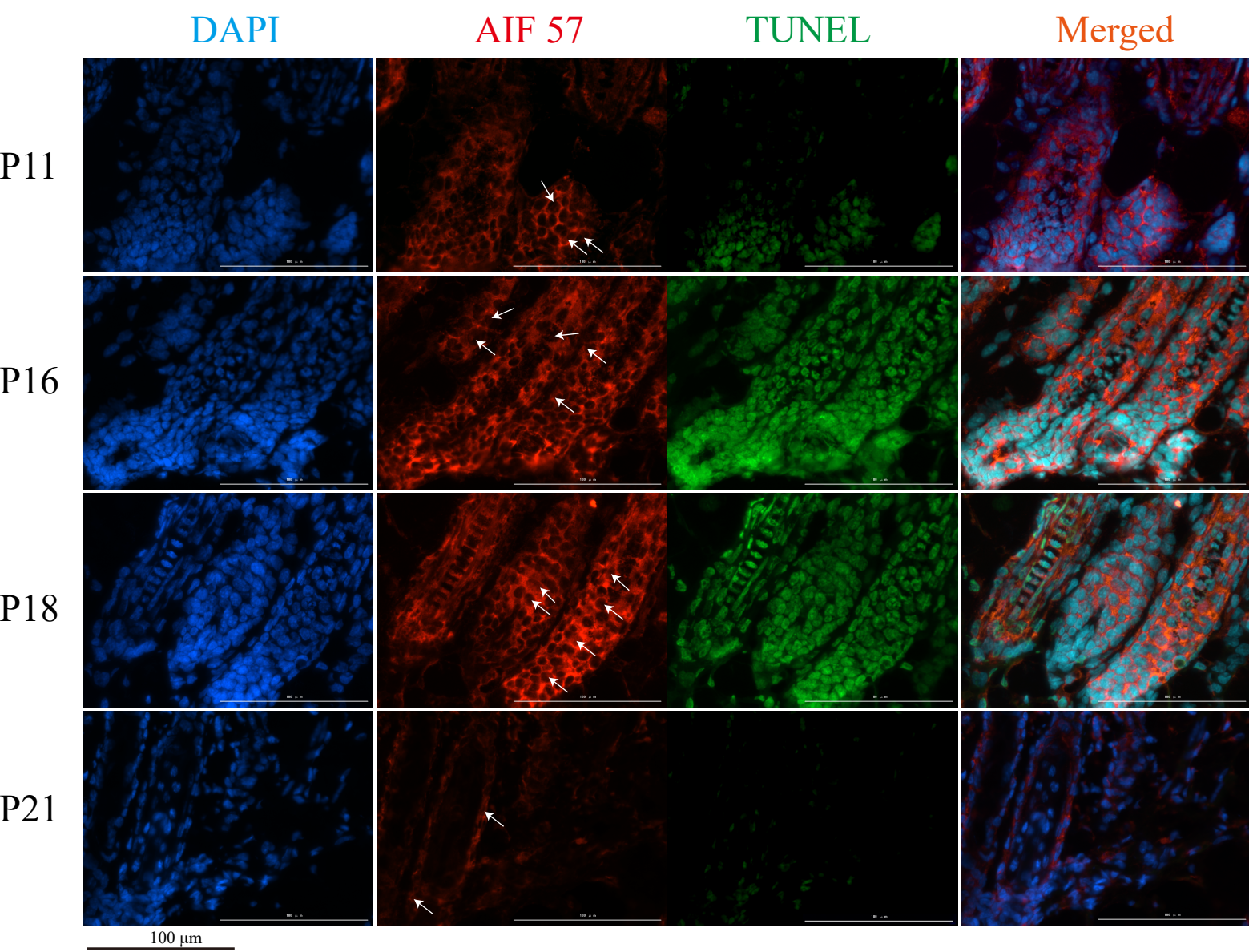

100  $\mu$ m

Supplement: Supplementary 3 — Figure S1: HE staining of skin at different stages of the hair cycle. Figure S2: Ki67 and TUNEL costaining of skin at different stages of the hair cycle. Figure S3: AIF-57 and TUNEL costaining of skin at different stages of the hair cycle (enlargement of the inset images in Figure 2(a)). [file 8279269.f3.zip › Figure.s3.pdf]
